# Disposable FFP2 and Type IIR Medical-Grade Face Masks: An Exhaustive Analysis into the Leaching of Micro- and Nanoparticles and Chemical Pollutants Linked to the COVID-19 Pandemic

**DOI:** 10.1021/acsestwater.1c00319

**Published:** 2022-03-23

**Authors:** J. Delgado-Gallardo, G. L. Sullivan, M. Tokaryk, J. E. Russell, G. R. Davies, K. V. Johns, A. P. Hunter, T. M. Watson, S. Sarp

**Affiliations:** † SPEC, College of Engineering, Swansea University, Swansea SA2 8PP, U.K.; ‡ SPECIFIC, College of Engineering, Swansea University, Swansea SA2 8PP, U.K.; § Advanced Imaging of Materials Facility, Bay Campus, College of Engineering, Swansea University, Swansea SA1 8EN, U.K.; ∥ Technical Development Center Analytical Laboratory, Tata Steel Europe, Harbourside Business Park, Port Talbot SA13 1SB, U.K.; ⊥ National Mass Spectrometry Facility, Swansea University Medical School, Singleton Park, Swansea SA2 8PP, U.K.

**Keywords:** microplastics, nanoplastics, heavy metals, COVID-19 PPE, disposable masks, medical-grade masks

## Abstract

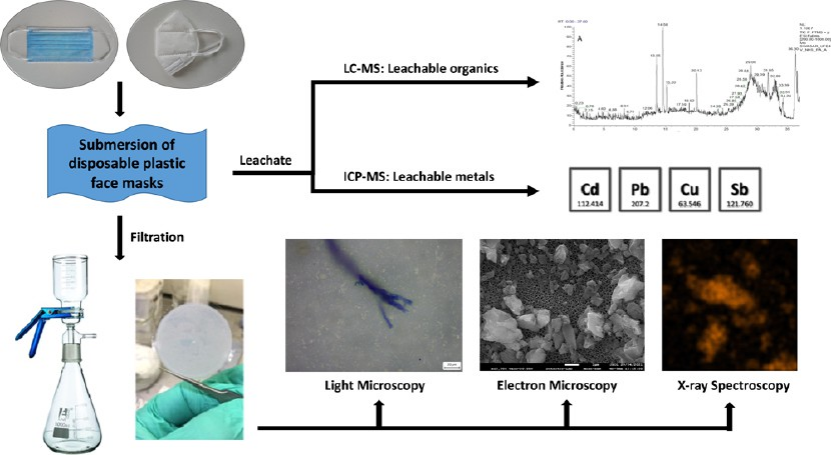

The COVID-19 pandemic has increased the worldwide production and use of disposable plastic face masks (DPFMs). The release of micro- and nanopollutants into the environment is one of the impacts derived from regulated and unregulated disposal of DPFMs. This study focuses on the emission of pollutants from medical-grade DPFMs when submerged in deionized water, simulating regulated and unregulated disposal of these masks. Three brands of FFP2 and three brands of Type IIR medical masks, produced in various countries (UK, EU, and non-EU), were investigated. Field emission gun scanning electron microscopy (FEG-SEM) was used to obtain high-resolution images of the micro- and nanoparticles, and 0.02 μm pore size inorganic membranes were used to retain and subsequently analyze smaller particle size nanoparticles (>20 nm) released from the DPFMs. Particles and fibers in the micro- and nanoscale were found in all six DPFM brands. SEM with energy-dispersive spectroscopy analysis revealed the presence of particles containing different heavy metals like lead, mercury, and arsenic. Inductively coupled plasma mass spectrometry analysis confirmed the leaching of trace heavy metals to water (antimony up to 2.41 μg/L and copper up to 4.68 μg/L). Liquid chromatography–mass spectrometry analysis identified polar organic species related to plastic additives and contaminants such as polyamide-66 monomers and oligomers.

## Abstract 2

The leaching of heavy metals and nanosize particles into the environment from medical-grade DPFMs is the focus of this article.

## 1 Introduction

The SARS-CoV-2 pandemic has increased the worldwide use of disposable plastic face masks (DPFMs) to safeguard the population’s health. The wearing of face coverings has been made mandatory in various settings in an effort to protect the general population.^1^ Because of the subsequent increase in demand, the production rates for masks in the UK have been increased by setting up several new, or improved, manufacturing facilities.^2,3^ Similarly, in Europe, the production capacity has increased 20-fold, compared to prepandemic levels, to 1.5 billion masks a month.^4^ This increment in face mask usage has led to a surge of DPFMs entering the marine environment, contributing to greater pollution of these bodies of water^5^ in the form of macro-, micro-, and nanoplastics (NPs).

There are a variety of types of disposable masks that differ based on their use and classification under relevant standards. Two such types designed for medical use are FFP2 and Type IIR, which are classified as a respirator mask and as a surgical mask under the British Standards BS EN 149:2001 + A1:2009 and BS EN 14683:2019, respectively. These two types offer different forms of protection: FFP2 protects the wearer from infection, while Type IIR is intended to protect the wearer’s surroundings.^6^ As they offer greater protection, FFP2 masks are recommended for use within high-risk areas, such as in hospitals,^7^ while Type IIR masks, although still used in medical applications, are the more common of the two types used among the general population.^8^ In addition, under normal circumstances, both designs of masks would be required to obtain CE marking (approved to be sold in the European Economic Area) before sale within the European Economic Area and the UK to show that they are safe and legal.^9^ However, to prevent delays in new products entering the market during the pandemic, some products have been authorized for sale without this assessment and markings.^10^ Different brands of masks of the same design may display different CE numbers depending on which notification body is used for the assessment of the masks.^11^

Even when businesses and organizations usually have a standard procedure for the disposal of DPFMs, a great amount of them are disposed incorrectly by the general public. DPFMs are potentially a source of microplastics (MPs) and NPs as well as other secondary contaminants.^12^ The secondary pollutants that may be released are of particular concern as the compounds used can pose significant risks to the environment.^13^ Elements like arsenic, lead, mercury, and antimony, among others were found as part of the particles released into water by the DPFMs selected in this study.

MPs and NPs are formed, among others, after the degradation of single-use plastic products, like disposable face masks (referred to as secondary MPs) by ultraviolet (UV) light or mechanical means.^14^ In addition, there is also the potential for particles to be released directly, without the need for material breakdown, as research has shown that masks can have a significant amount of loosely attached plastic particles.^15,16^

Recent studies have pointed at the contribution of DPFMs, via direct disposal or by laundering, to the aquatic plastic pollution during and beyond the pandemic.^17,18^ This contamination also extends to soil^19^ and ultimately to wildlife.^20^ Research has shown that both MPs and NPs can have toxic effects if they are ingested and can be transferred along the food chains by ingestion and bioaccumulation and have effects on the development and reproduction of the organisms by interfering with their metabolism.^21^ Furthermore, these particles can adsorb contaminants already present in the water and transport them, if they are ingested, into marine animals, leading to an increased build-up of harmful compounds within the animals.^22^ A recent study has also investigated the risk of particle inhalation posed by wearing DPFMs.^23^

Determining which plastics and other pollutants are released from these FFP2 and Type IIR masks when they are submerged in water can then help establish the wider impact their use will have on the environment. The current studies found on this topic used Fourier transform infrared (FT-IR) spectroscopy to determine the plastics used within respirator and/or surgical masks. These studies revealed plastics that are known to form nanoparticles, but the research did not generate or analyze any NPs or other contaminants released from the masks.^24−26^

This study incorporates field emission gun-scanning electron microscopy (FEG-SEM) and uses 20 nm pore size inorganic membranes in order to retain and analyze particles below 100 nm, which is widely accepted as the boundary of the nanoscale. The nanoscale is defined between 1 and 100 nm,^27^ and it marked the size limit described by the authors of previous research.^16^ The study focuses exclusively on medical DPFMs manufactured in various countries (UK, EU, non-EU), including a Type IIR brand, which was used by the UK National Health Service (NHS).

Several technologies, including SEM, inductively coupled plasma mass spectrometry (ICP-MS), and liquid chromatography–mass spectrometry (LC–MS), were used to detect and characterize organic and inorganic pollutants. Some concerning heavy metals (i.e., mercury) previously undetected were identified along with a vast range of other components.

## 2 Methodology

### 2.1 Leaching and Separation of Particles

**Figure 1:**
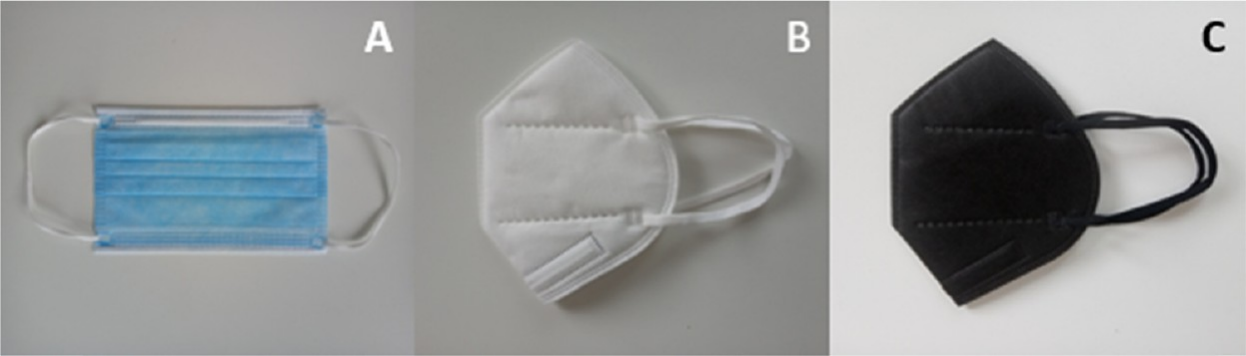
Images of masks used for this investigation. (A) Omnitex (showed as an example of all three Type IIR DPFMs), (B) Baltic (same features as Soyes), and (C) Geji (both FFP2 type).

For this study, a selection of six different brands of DPFMs, three FFP2 and three Type IIR (Figure 1), from different manufacturers (Table 1), were acquired from Amazon. The FFP2-type masks used were sold under the brands Baltic, Soyes Geji (referred to as Geji within this paper), and Soyes. While the Type IIR brands were Omnitex, Duronic, and ones certified for use in COVID-19 test centers, and other environments, by the NHS. All the masks used were confirmed to conform to the relevant British Standard.

**Table 1:** Manufacturer and Distributor Information for DPFMs Used in the Experiment

brand	type	manufacturer
Baltic	FFP2	InSpe (LT)
Duronic	II-R	Wujiang Kangjie Medical Co., Ltd. (CN)
Geji	FFP2	Guangzhou Dingdun Technology Co., Ltd. (CN)
NHS	II-R	Anhui JBH Medical Apparatus Co., Ltd. (CN)
Omnitex	II-R	Sharon Services (UK) Ltd.
Soyes	FFP2	Zhangzhou Anyue Sanitation Supplies Co., Ltd. (CN)

For each batch of experiments, three sets of three of the same brand of mask were placed into beakers with 1 L of deionized water, dispensed from a Milli Q type 1 dispenser. The masks were left submerged for 4 h at room temperature with gentle agitation using a glass stirrer, once an hour. The same procedure was repeated with a beaker containing only deionized water to be used for blank/control tests.

After submersion, the leachate was filtered through either a 0.1 μm or a 0.02 μm Al_2_O_3_ Whatman Anodisc membrane under vacuum using an Aldrich glass funnel and receiving flask. Three repetitions were conducted using each membrane pore size for each brand of DPFMs. Once all the leachate from a beaker had been filtered, the membrane was placed within a glass Petri dish and allowed to dry at room temperature overnight. Using a beaker containing only deionized water, a blank membrane was also treated using the same filtration method as described above.

### 2.2 Microscopy of Leached Particles

Light microscopy of the membranes was used to determine the coverage of particle contamination, and this was done using a Zeiss Primotech microscope (Carl Zeiss Ltd., Cambridge, UK) at 50× and 500 overall magnification.

For SEM and energy-dispersive X-ray spectroscopy (EDX) analysis, a Tabletop Microscope TM3000 was utilized (Hitachi High-Technologies Corporation), and samples were mounted on carbon tape and placed in a vacuum chamber.

A new technology was also put in place. Furthermore, high-resolution characterization was conducted using a secondary electron detector on a JEOL 7800F field emission gun scanning electron microscope (JEOL, Tokyo, Japan). Prior to imaging, these samples were coated in 10 nm of platinum.

### 2.3 ICP-MS Elemental Analysis for Heavy Metals in Leachates

Three DPFMs from each brand (labeled; Baltic, Duronic, Geji, NHS, Omnitex, and Soyes) were submerged in 250 mL of deionized water and left for 24 h. The aqueous portion (50 mL) of the previous sample solution, termed the “leachate,” was then aliquoted into clean centrifuge tubes and acidified using 1 mL of 1 M nitric acid. The leachates were analyzed for metal impurities using a Perkin Elmer ICP-MS NexIon 2000, a common method for elemental analysis in aqueous samples. A procedural blank (deionized left in glass container for 24 h) and reagent blank (deionized water only) were run with samples to check for background interference. To ensure there was no carryover, blanks were run after the highest calibration and after every sample. Calibration curves for each heavy metal tested can be found in the Supporting Information (Figures 46S–58S). QC10, LOD, and procedure blank analyses are also supplied in the Supporting Information (Table 1S).

A calibration standard, containing a mixture of heavy metals (As, Cd, Cr, Co, Cu, Mo, Ni Pb, Sb, Ti, and Hg) was acidified using 10% HNO_3_ and diluted to a calibration range of 1 to 20 μg/L. An external calibration curve was generated from the injection of the diluted standards, and it required regression statistics above 0.9990 to be deemed acceptable. The samples that were concentrated above 20 μg/L were further diluted using acidified (10%) deionized water to bring into the dynamic range of the method. A number of quality control samples, prepared from a separate batch of multielement standards and run at the midrange of the method (10 μg/L), were put in place to assess the accuracy and precision of the analytical method, and they were required to be below 10% for both to be deemed acceptable. The instrument parameters were optimized prior to running samples and were set using the following conditions: plasma gas flow set at 18 L/min of argon, auxiliary gas flow set at 1.8 L/min, and nebulizer flow rate set at 0.98 L/min. The sample uptake was set at 300 μL/min with three replicates per sample. The radio-frequency voltage was applied at 1600 W, as determined by method optimization during method development.

### 2.4 LC–MS Screen for Organic Compounds in Leachates

As shown in Section 2.3, three DPFMs from each brand were placed in 250 mL of deionized water and left for 24 h. The leachate was removed and analyzed using reverse-phase high-pressure liquid chromatography (HPLC), with positive electrospray mass spectrometry (ESI-MS). No sample pretreatment was performed on the leachate. Five microliters of the sample were loop-injected into the pretuned and calibrated LC/MS system. The system blanks and procedural blanks were run to identify the background ions and assess any potential carryover.

Organic contaminants were identified using a Thermo LTQ Orbitrap XL accurate mass spectrometer based on the empirical formula deduced from the accurate mass calculations and isotope patterns. For chromatographic separation, a reverse-phase XBridge C18 column, with dimensions 3.5 μm × 2.1 mm × 150 mm, and a Guard column: XBridge C18, 3.5 μm × 2.1 mm × 10 mm, were used for analyte separation, with a flow rate of 150 μL/min for LC/MS. The composition of the mobile phase A was 0.1% formic acid; 2% (acetonitrile) MeCN in (water) H_2_O, and the mobile phase B was 0.1% formic acid in MeCN. The elution gradient started at 2% B and increased to 90% at 32 min before returning to 2% B and ending with a total run time of 37 min.

A Dionex Ultimate 3000 HPLC system was connected in series to a Thermo LTQ Orbitrap XL accurate mass spectrometer, with an API ion spray source. The MS parameters were optimized for a typical screening method. The following conditions were used: sheath gas flow was set to 15 L/min, auxiliary gas flow was set at 2 L per minute, and the probe voltage was set at 4.3 kV with a capillary and tube lens voltage of 43 and 150 V, respectively. The MS scan conditions were full mass profile mode *m*/*z* 200–1000 with a resolution of 60,000 and a mass error of 0.5 ppm.

## 3 Results and Discussion

### 3.1 Optical Microscopy of Membranes

**Figure 2:**
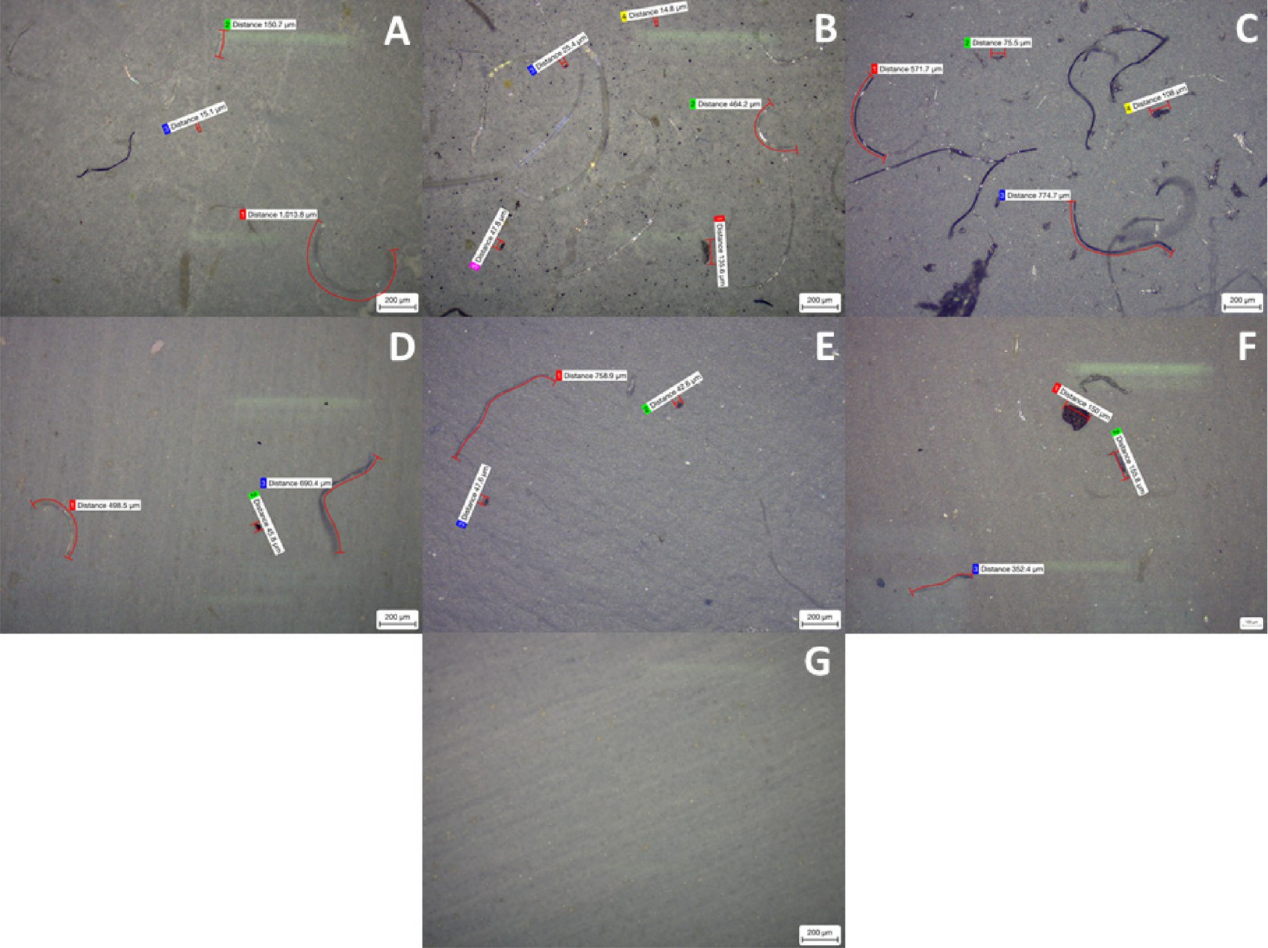
Light microscopy images at 50× overall magnification of 0.02 μm pore size membranes for all brands after filtration. (A) Baltic, (B) Geji, (C) Soyes (all FFP2), (D) Duronic, (E) NHS, (F) Omnitex (all Type IIRR), and (G) blank.

Light microscopy analysis revealed that all the masks tested emitted fibers visible at 50× overall magnification. The colors of the fibers seen were in line with the visual appearance of the masks they originated from, namely, white and blue fibers from the Type IIR masks, white from the Baltic and Geji masks, and black from the Soyes masks. Figure 1S (see the Supporting Information) and Figure 2 below show the microscopy images for 0.1 and 0.02 pore size membranes, respectively, for each of the brands and the blank sample at 50× magnification. More images of the replicates can be found in the Supporting Information (Figure 4S–31S). All three of the FFP2 masks emitted significantly greater amounts of microplastic fibers and particles than all Type IIR masks. Optical microscopy image counting of areas of 3 μm^2^ gave four to six times higher particle amounts released from FFP2 masks than Type IIR masks. This result contravenes the findings of previous research on the inhalation of MPs in air which found that N95 masks, which are approximately equivalent to FFP2 masks, emitted lower amounts of fibers than Type IIR masks.^23^ The difference found in this work may be due to the fact that the fiber structure of melt-blown fabrics, which make up the inner layers of both designs of masks as stated in the information provided by the manufacturers, is known to be damaged during washing with water.^23^ As FFP2 masks have three inner layers,^28^ compared to the single layer in Type IIR masks,^26^ there is a larger amount of fabric to be damaged in this design of mask, potentially leading to a greater number of emitted fibers.

**Figure 3:**
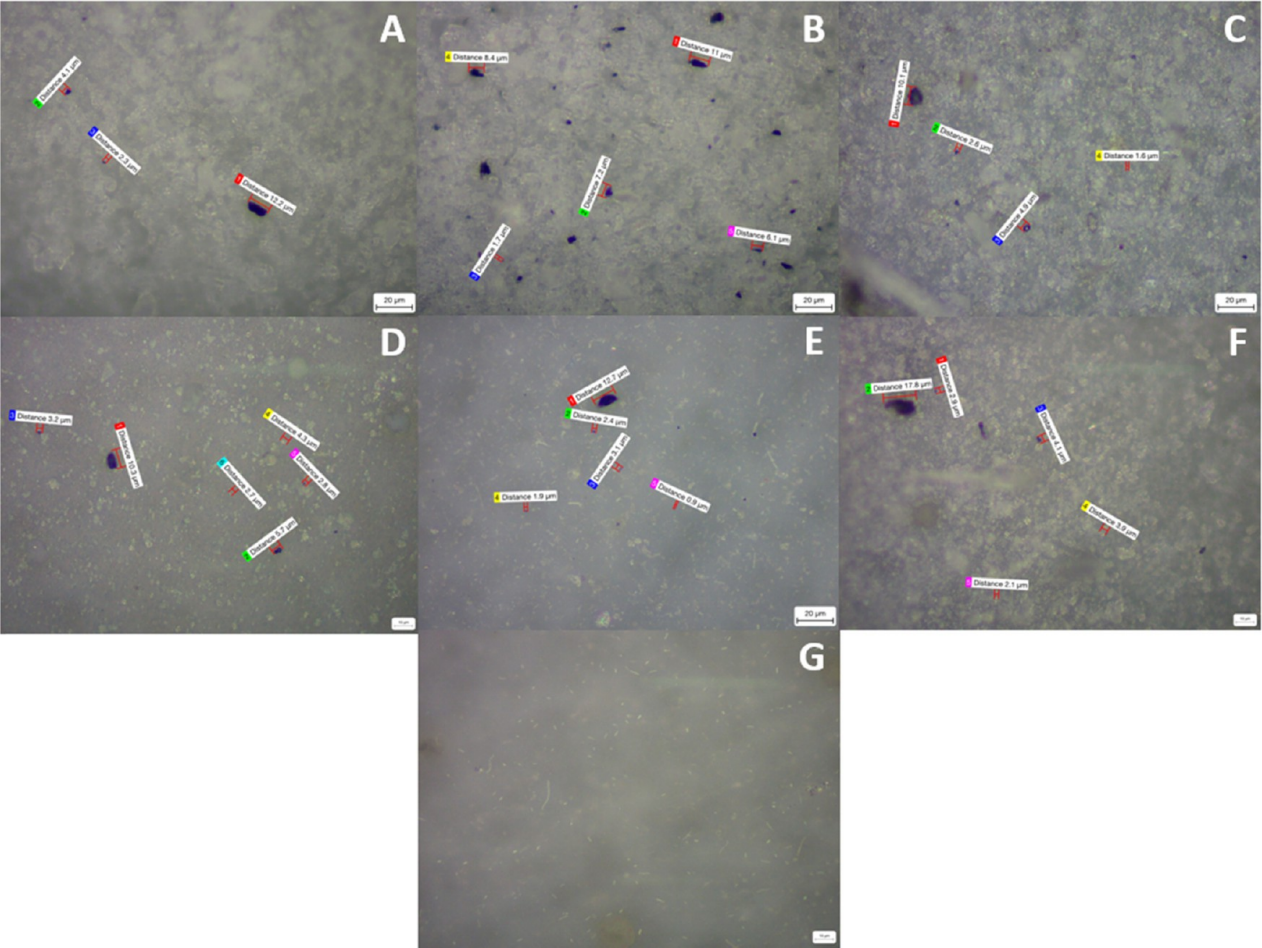
Light microscopy images at 500× overall magnification of 0.02 μm pore size membranes for all brands after filtration. (A) Baltic, (B) Geji, (C) Soyes (all FFP2), (D) Duronic, (E) NHS, (F) Omnitex (all Type IIRR), and (G) blank.

In addition to the fibers, all the mask brands also emitted smaller fragments that were retained by the membranes, some of which required 500× overall magnification. These particles ranged in size from around 100 μm to approximately 1.5 μm, which aligns with the size of microplastic particles (<1 mm).^29^ The number of these fragments detected was limited for all brands, except for the membranes used to filter the Geji mask leachates. This can be seen in Figure 2S (see the Supporting Information) and Figure 3 below, which shows the microscopy images from both 0.1 and 0.02 pore size membranes, respectively, taken at 500× magnification, for each brand and the blank sample.

As the number of fragments emitted from the other FFP2 masks was similar to the amount released by the Type IIR masks, a large number of fragments from the Geji masks were thought to be likely due to the foam nose cushions, which were only present on this brand of mask. Foams of this type in the microplastic form are known to have effects on microbial ecosystems in marine sediment,^30^ as well as leaching harmful additives.^31^ As such, these fragments pose a similar risk to the environment as the other fragments and fibers released by the masks; however, some strains of bacteria and fungi have been reported as being able to efficiently degrade the foams.^32^

### 3.2 Analysis of Micro- and Nanoparticles with SEM

**Figure 4:**
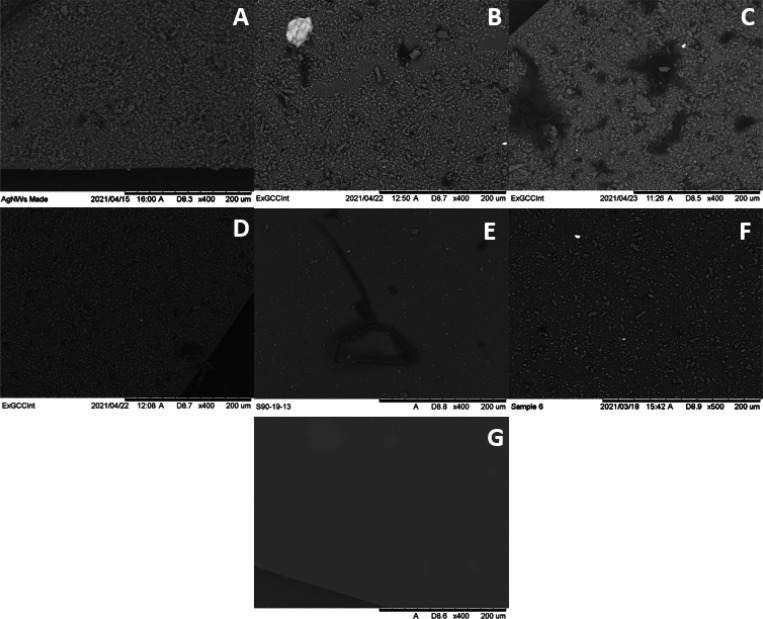
SEM images at ×400 for all 0.02 μm membrane surfaces after filtration. (A) Baltic, (B) Geji, (C) Soyes (all FFP2), (D) Duronic, (E) NHS, (F) Omnitex (all Type IIRR), and (G) blank membranes (only deionized water was filtered).

The images taken using SEM techniques show the deposition of fibers and particles in all membranes. The particles with lighter colors usually contain heavier elements. The difference between the mask leachates and the blank membranes, which were filtered with deionized water and left for 4 h and stirred, the same as the other samples, is notable, as the number of particles is clearly much lower on the blanks. Apart from that, the NHS brand was the one with a lower number of particles on the surface (Figure 4).

This difference in the number of particles deposited between blank membranes and the rest was confirmed by the second FEG-SEM. Figure 3S (see the Supporting Information) and Figure 5 show results for 0.1 and 0.02 pore size membranes, respectively. More images of the replicates can be found in the Supporting Information (Figures 32S–45S).

**Figure 5:**
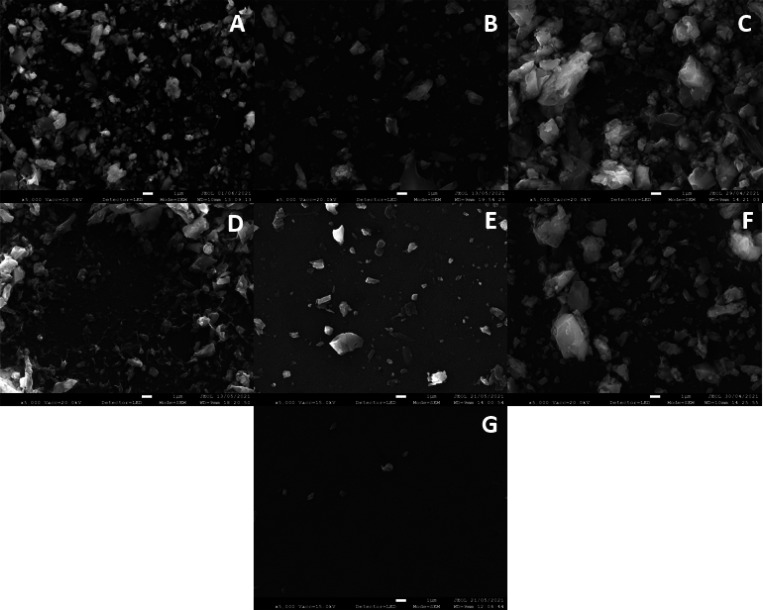
FEG-SEM images of 0.02 μm pore size membranes for all brands at ×5000. (A) Baltic, (B) Geji, (C) Soyes (all FFP2), (D) Duronic, (E) NHS, (F) Omnitex (all Type IIRR), and (G) blank.

**Figure 6:**
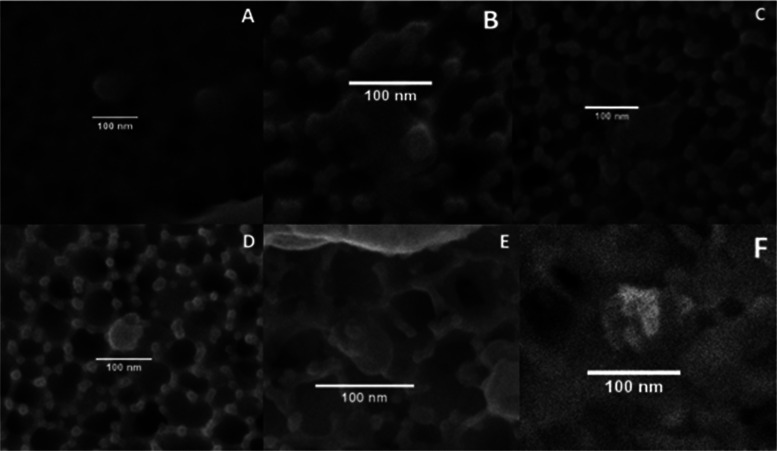
SEM images of nanoparticles found on the membrane surfaces after filtration. (A) Baltic, (B) Duronic, (C) Geji, (D) NHS, (E) Omnitex, and (F) Soyes.

The presence of nanoparticles (>100 nm) was also confirmed in all brands (Figure 6).

### 3.3 SEM with Energy-Dispersive Spectroscopy Elemental Characterization of Particles

The composition of the particles and element traces found in all brands and blanks are detailed as follows, and the presence of these traces is shown in Table 2. Traces of diverse elements and heavy metals like antimony, arsenic, cadmium, chlorine, iron, lead, mercury, nickel, platinum, silicon, tin, and titanium, among others, were found in all brands of this study, to a greater or lesser extent (Figures 59S–61S in the Supporting Information). The sorption and subsequent desorption of metals and metalloid cations by MPs have been proven, emphasizing the relevance of these pollutants as vectors and also revealing how smaller particles have a higher accumulation of metals.^33^

**Table 2:**
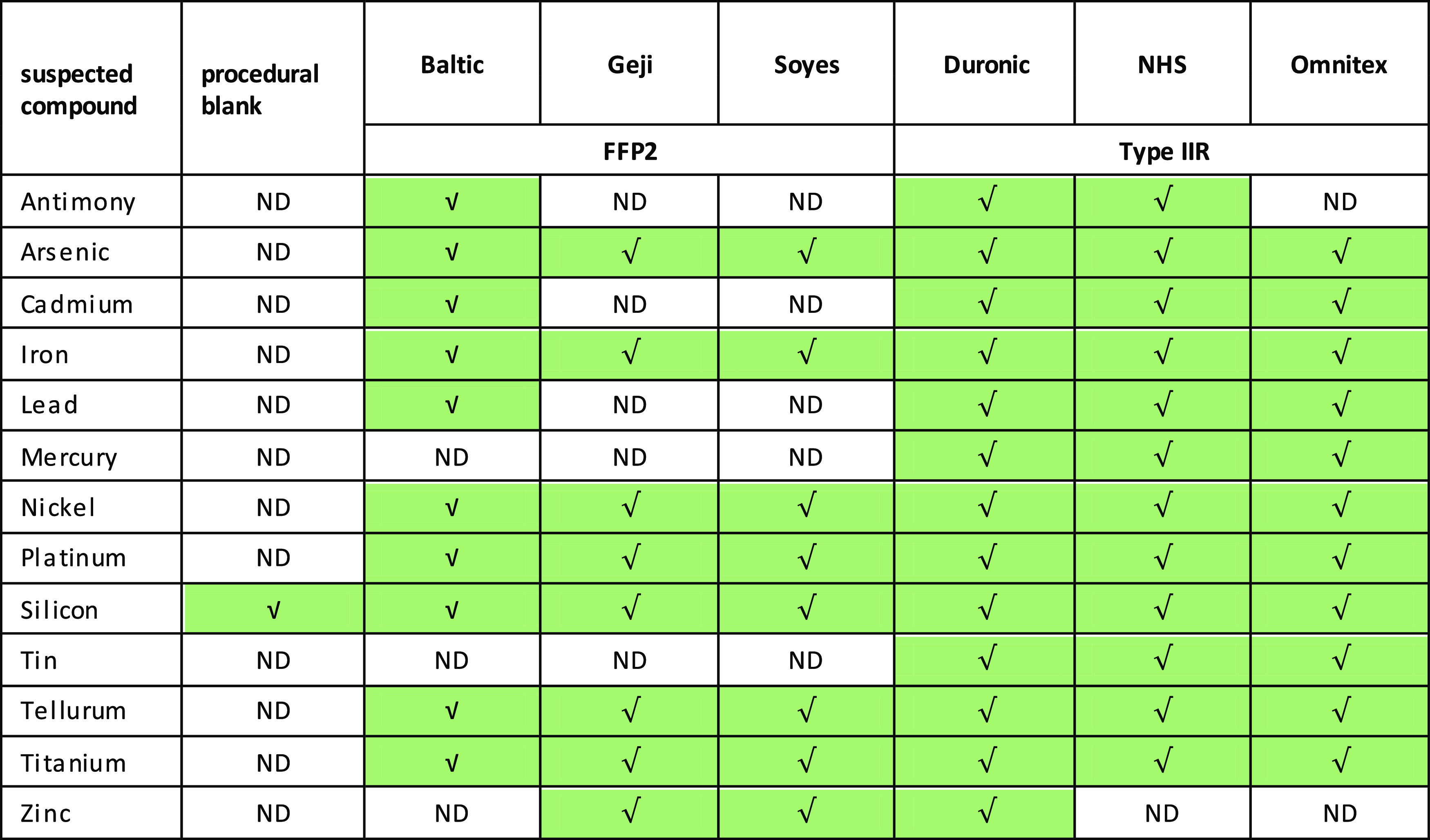
Different Chemical Elements Found in Particles Deposited on the Inorganic Membranes after Filtration^a^

a √ Refers to the presence of the element.

These heavy metals can have several different effects, depending on the specific metal and its concentration and speciation, including neurological disorders and muscular diseases.^34^ In addition, some masks have titanium dioxide nanoparticles bound within the fibers, as this compound exhibits antimicrobial properties.^35,36^ Research has shown that such nanoparticles can cause oxidative stress and have a genotoxic effect.^37^

**Figure 7:**
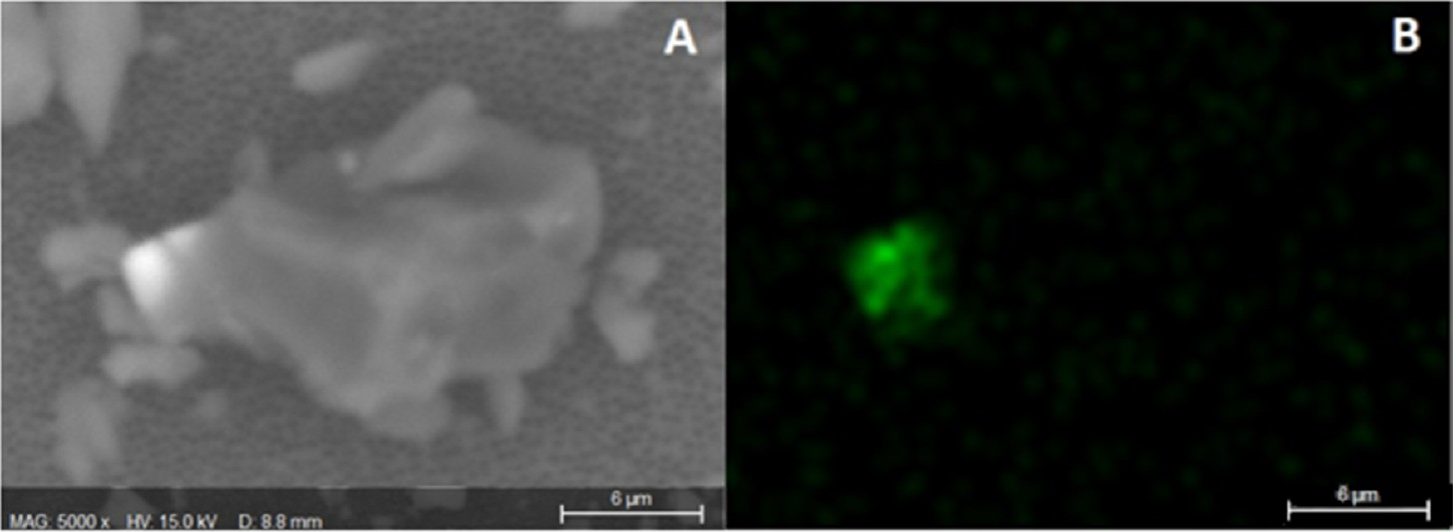
Particle on membrane containing Fe. (A) SEM-generated image, (B) false color map for elemental Fe.

**Figure 8:**
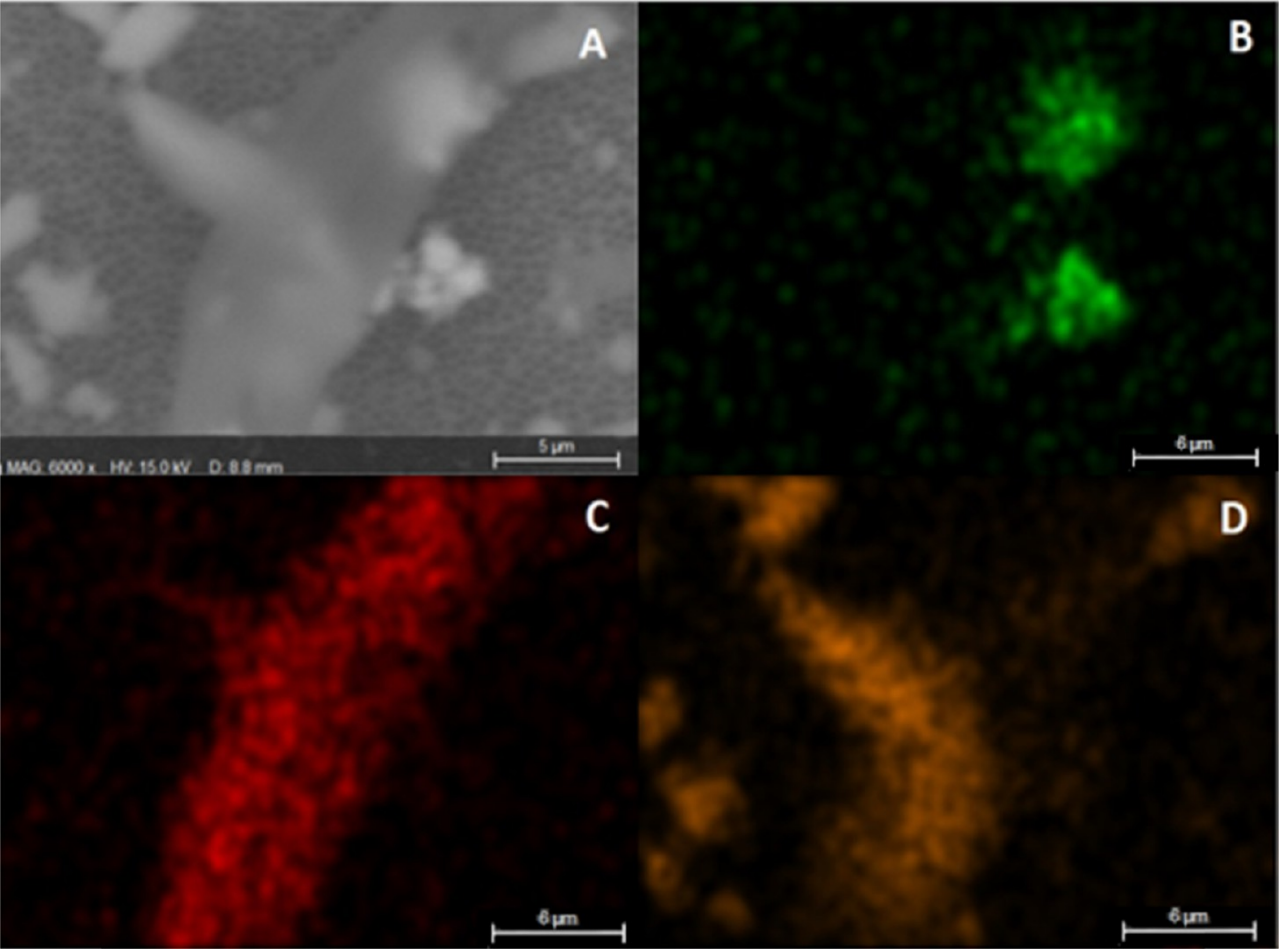
Fiber on membrane containing Fe and Si. (A) image generated by the SEM, (B) false color map for elemental Fe. (C) false color map for elemental C and (D) false color map for elemental Si.

Particles containing high percentages of iron (Fe) have been observed as part of larger particles (Figure 7) and as part of fibers. The fibers also contained Si on their structure (Figure 8). DPFMs usually have nose strips that may be made of steel, and possibly PVC/PE.

Complex particles with high percentages of Fe and traces of many different heavy metals were found in NHS samples. The elemental scanning of one of these particles gave concentrations of 3.65% of As, 3.47% of Cd, 3.73% of Cu, 4.71% of Hg, 3.96% of Ni, 5.65% of Pb, and 4.92% of Sn, among others. Similar compositions were found in particles of the same characteristics (Figure 62S in the Supporting Information).

The particles found after filtration of the blank samples presented mostly compositions of silica with other elements in low concentrations (like 0.29% of Cl), but not as complex as the ones found with the submersion of DPFMs, and no heavy metals were detected. The number of particles found was significantly lower than the leachates that had DPFMs submerged (Figures 4 and 5).

### 3.4 ICP-MS Results

For ICP-MS analysis of samples, a full external calibration was performed to determine the analyte concentration, and reagents and procedural blanks were used prior to and after analysis of the samples to assess any potential carryover. All QC determinants for As, Cd, Cr, Co, Cu, Mo, Ni Pb, Sb, Ti, and Hg passed the acceptance criteria. For blank samples, all values possess concentration values below the analytical detection limits, except for Cu, Mo, Pb, and V. Subsequently, any positives in samples were blank-subtracted and -corrected. Table 3 shows the leachable heavy metals from the DPMFs (converted to 1 mask per liter). Three masks were added to 250 mL of deionized water. To convert values to 1 mask per liter, the instrument values (Table T1S) were blank-corrected and then divided by a factor of 12.

**Table 3:** Results of Metals Leached from Medical DPFMs in μg/L^a^

sample identity	As	Cd	Co	Cr	Cu	Mo	Ni	Pb	Sb	Ti
	μg/L									
Geji	ND^b^	ND	ND	0.015	0.200	0.083	0.018	0.005	0.312	0.001
Duronic	ND	0.001	ND	ND	0.201	0.006	0.015	0.001	0.011	0.001
Baltic	ND	ND	0.003	0.029	0.093	0.003	ND	ND	2.413	0.001
Omnitex	ND	0.001	ND	ND	4.676	>0.03	0.025	0.005	0.373	0.002
Soyes	ND	ND	ND	0.033	0.033	>0.03	0.006	ND	0.149	ND
NHS	ND	ND	ND	ND	0.065	>0.03	0.007	ND	ND	0.001

a Raw data can be seen in the Supporting Information with the LOD and calibration curves.

b ND, not detected (below the detection limit, please see the Supporting Information Figures 46S–58S).

The metal levels found in the DPFM leachates were all typically low levels, the majority being subparts per billion (ppb) levels, and some not detected altogether (As and Hg). The highest metal determinates were found to be Cu (Omnitex mask leachate at 4.68 μg/L) and Sb (Baltic mask leachate at 2.41 μg/L).

Overall, FFP2 DPFMs appear to have significantly lower levels of trace metals than that of recently studied Type IIR masks analyzed in the previous publication. Upon the comparison of Sb and Pb levels present in FFP2 DPFM leachates and Type IIR DPFMs, Sb ranged from 0.01–2.41 μg/L and Pb >0.001–0.005 μg/L in FFP2 type, whereas in Type IIR, it had concerning levels of Sb ranging from 27.8–98.25 μg/L, and Pb values up to 1.70 μg/L, indicating a significant increase in emission in Type IIR DPFMs (values converted from a previous publication to 1 mask per liter by dividing values by 4).

Again, analysis for the emission of toxic metals Cd, Ni, and Cr proved negligible for the DPFMs in this study. Cd was found at very low parts per trillion (ppt) level in Duronic and Omnitex brand leachates, with a slight elevation of chromium detected in Geji, Soyes, and Baltic samples (0.015–0.033 μg/L). Nickel was found in all masks apart from Baltic, likely to arise from the metal wiring parts in the mask, but this was still at very low levels (0.006–0.025 μg/L).

### 3.5 LC–MS Results

**Figure 9:**
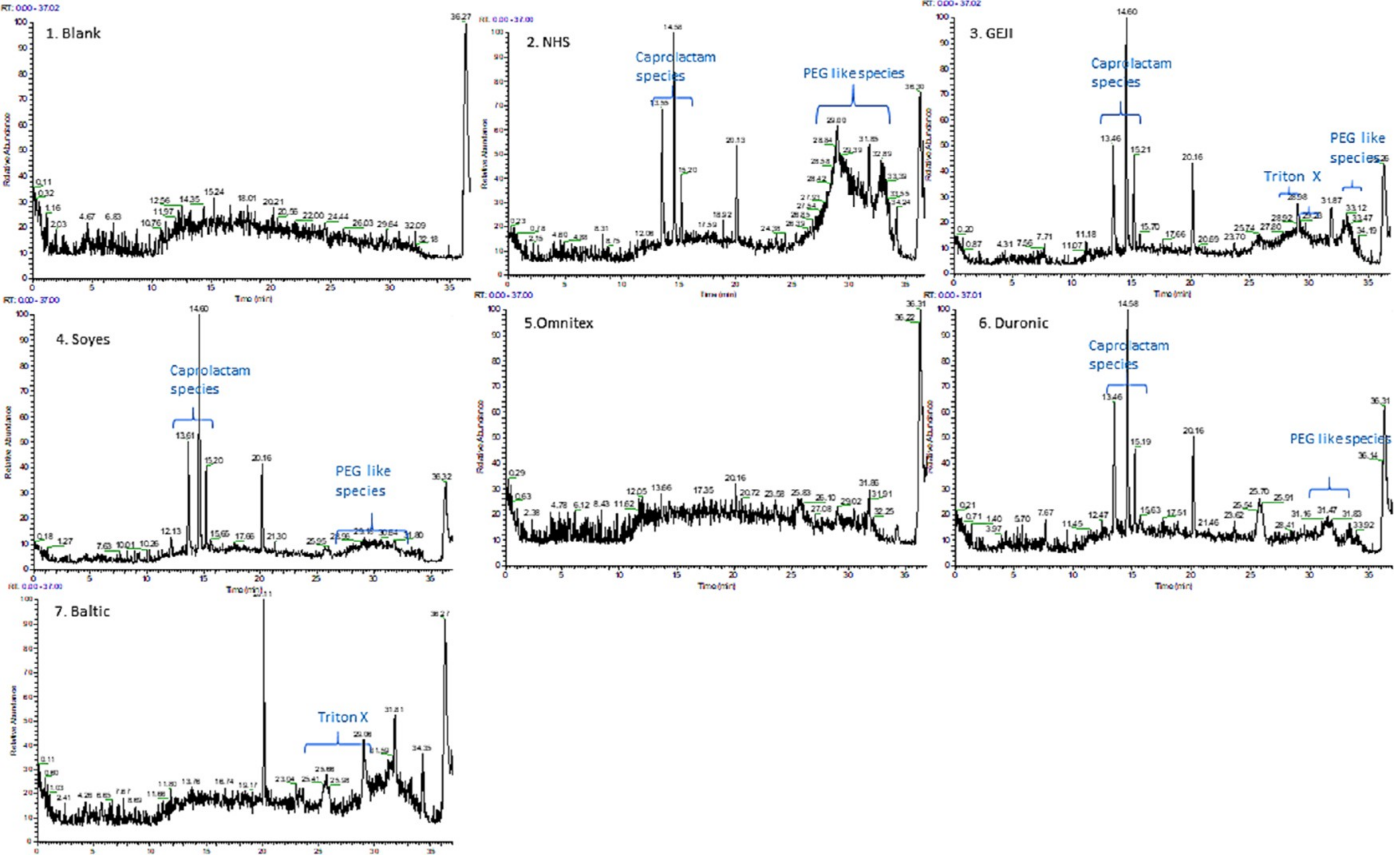
Total ion chromatogram (TIC) (1–7) from the LCMS analysis of the leachate of all samples and blank. Omnitex (5) appears to release the least number of organic species and its TIC has a similar appearance to that of the blank sample (1). Caprolactam polymeric species, PEG polymers, and detergent molecules have been labeled on associated chromatograms.

The organic contaminates present in the leachate were assigned identity based on accurate mass data, ms/ms fragmentation information, and analyst discrimination. Polymeric species of caprolactam, −(C_6_H_11_NO)_*n*_ (a precursor for nylon 6 or 66 with peaks between 7.5 and 9.5 min [*m*/*z* (209), 227, 249], 13.5 min [*m*/*z* 322,340 and 362], 14.6 min [*m*/*z* 435, 453 and 475], 15.3 min [*m*/*z* 548,566, and 588], and 15.7 [*m*/*z* 679/701]), was found in brands Duronic, Geji, NHS, and Soyes. These were not identified in Baltic, Omnitex, and blank samples (Table 4 and Supporting Information Tables T1S and T2S and Figure 9).

**Table 4:**
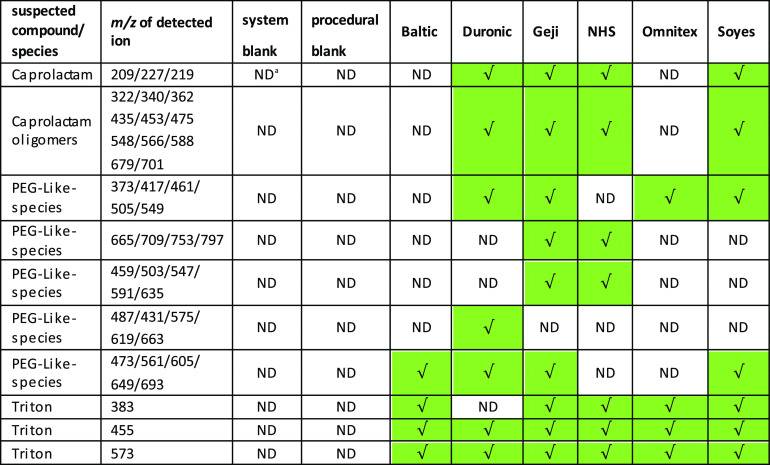
Leachable Organic Compounds from DPFMs Identified by LC–MS Accurate Mass; ND Refers to Analyte Not Being Detected, √ Indicates Its Presence^a^

a ND, not detected.

Ions consistent with polyethylene glycol (PEG) polymers (homologues series separated by *m*/*z* 44) and triton-reduced (*m*/*z* 455, 527) nonionic surfactants were observed in all sample leachates but not in the blanks, indicating evidence of organic substances being released from the DPFMs. Similar organic compounds were present in Type DPFMs analyzed in a previous study.

PEG-like and detergent compounds are likely to be associated with plasticizers, applied to modify the plastic properties during the manufacture of the DPFM plastic materials, while the caprolactam oligomers originate from nylon-6,6 precursors, used in the manufacture of the elasticated parts of the masks.^38,39^ All these organic leachable compounds, commonly found in the textile industry, have known environmental impacts associated, and thus, the release from DPFMs is an additional cause of concern.^40,41^

Also, the nonionic surfactant Triton X was detected. In 2012, this detergent was included in the “List of substances of very high concern” of the Registration, Evaluation, Authorization, and Restriction of Chemicals by the European Chemicals Agency, mandating industries, including pharmaceuticals, its replacement by January 2021.^42^ One of the products resulting from the degradation of Triton X has been demonstrated to have hormone-like properties that might affect organisms in the environment, and thus, is also a cause of concern.^43^

Other compounds, like PEG, were present after LC–MS analysis. Although PEG is considered biologically inert, approximately 72% of the population has been proved to have PEG antibodies.^44^ Because of this, allergies and hypersensitive reactions are an increasing concern.^45,46^

## 4 Conclusions

FFP2 and Type IIR medical-grade DPFMs have been shown to release micro and NP particles and fibers when submerged in water. Characterization of the masks determined that these particles and fibers were most likely made from polypropylene. Among the two designs of masks researched, FFP2 masks were found to emit more fibers than Type IIR masks with significant amounts of additional microplastic particles being released from foam nose cushions present on one of the FFP2 brand masks.

In addition to the plastic particles and fibers, the masks have also been shown to emit heavy metals (such as arsenic, antimony, mercury, lead, tin, and titanium), showing their potential as a serious contamination source because of their widespread use as a result of the COVID-19 pandemic. The levels of these pollutants are lower than those previously published for nonmedical DPFMs, and thus, medical DPFMs could present a lower risk. Nevertheless, more analysis should be performed in order to clarify the origin of these compounds and whether the manufacturing process must be revised and improved.

The presence of particles containing heavy metals in the masks is of particular concern as it is unknown how strongly they are bonded to the mask fibers. The potential leaching of these particles during the use of the masks, or when the masks are within the environment, requires further investigation. The bioaccumulation properties of the detected heavy metals are a cause of concern because of the great amount of medical DPFMs entering the environment when disposed. ICP-MS analysis results confirmed traces of heavy metals (antimony up to 2.41 μg/L and copper up to 4.68 μg/L). LC–MS analysis results identified polar leachable organic species related to plastic additives and contaminants, polyamide-66 monomer and oligomers (nylon-66 synthesis), surfactant molecules, and PEG.

These results claim for stricter regulations to be put in place regarding the manufacturing and disposal of DPFMs. Also, a complete investigation must be done to clarify the extent of the risks and the potential impacts of the fibers and particles released when using these items and when they enter the environment.

## Supporting Information Available

Detected instrument values, additional microscopy and SEM images, ICP-MS calibration curves, and additional EDX data (PDF)
