# Supplementary material for: Disposable FFP2 and Type IIR Medical-Grade Face Masks: An Exhaustive Analysis into the Leaching of Micro- and Nanoparticles and Chemical Pollutants Linked to the COVID-19 Pandemic
Source: ACS ES T Water. 2022 Mar 23;2(4):527–38. doi: 10.1021/acsestwater.1c00319 (PMC8982497; doi:10.1021/acsestwater.1c00319)
Supplement: Supplementary file 1 — ew1c00319_si_001.pdf [file ew1c00319_si_001.pdf]

**Disposable FFP2 and Type IIR Medical grade Face Masks - An exhaustive analysis into the leaching of micro and nanoparticles and chemical pollutants. Linked to the Covid-19 pandemic.**

J. Delgado-Gallardo[a], G. L. Sullivan[b], M. Tokaryk[a], J. E. Russell[c], G.R. Davies[d], K.V. Johns[d], A.P.Hunter[e], T.M. Watson[b], S.Sarp[a,*]

[a] SPEC, College of Engineering, Swansea University, SA2 8PP, UK.

[b] SPECIFIC, College of Engineering, Swansea University, SA2 8PP, UK.

[c] Advanced Imaging of Materials Facility, Bay Campus, College of Engineering, Swansea University, Swansea SA1 8EN, UK.

[d] Technical development center analytical laboratory, Tata Steel Europe, Harbourside Business park, , Port Talbot, SA13 1SB.

[e] National Mass Spectrometry Facility, Swansea University Medical School, Singleton Park, Swansea, SA2 8PP

*Corresponding Author: sarper.sarp@swansea.ac.uk

## Supporting Information

Table T1S. Shows the instruments values detected in leachate samples (3 masks in 250 mL deionised). To correct to 1 mask per L, values need to be divided factor of 12. Positive samples values are assigned if above the limit of detection after blank subtraction.

| Sample Identity | As | Cd | Co | Cr | Cu | Mo | Ni | Pb | Sb | Ti | V | Ag |
|---|---|---|---|---|---|---|---|---|---|---|---|---|
| | µg/L | | | | | | | | | | | |
| QC 10 | 10.137 | 10.005 | 9.990 | 10.219 | 9.875 | 10.424 | 10.314 | 9.973 | 10.353 | 10.573 | 9.698 | 9.911 |
| Blank | >0.13 | >0.008 | >0.009 | 0.210 | 0.171 | 0.307 | 0.610 | >0.008 | 0.312 | >1.0 | 0.017 | >0.009 |
| Geji | >0.13 | >0.008 | >0.009 | 0.176 | 2.400 | 0.226 | 0.216 | 0.061 | 3.744 | >1.0 | 0.010 | 0.009 |
| Duronic | >0.13 | 0.012 | >0.009 | >0.03 | 2.417 | 0.076 | 0.176 | 0.013 | 0.134 | >1.0 | 0.015 | >0.009 |
| Baltic | >0.13 | >0.008 | 0.038 | 0.349 | 1.110 | 0.031 | >0.07 | >0.008 | 28.961 | >1.0 | 0.012 | >0.009 |
| Omnitex | >0.13 | 0.012 | >0.009 | >0.03 | 56.117 | >0.03 | 0.304 | 0.629 | 4.481 | >1.0 | 0.023 | >0.009 |
| Soyes | >0.13 | >0.008 | >0.009 | 0.033 | 0.394 | >0.03 | 0.072 | >0.008 | 1.792 | >1.0 | 0.009 | >0.009 |
| NHS | >0.13 | >0.008 | >0.009 | >0.03 | 0.774 | >0.03 | 0.078 | >0.008 | >0.10 | >1.0 | 0.011 | >0.009 |
| QC 10 | 10.187 | 9.911 | 10.085 | 10.082 | 10.103 | 9.647 | 10.431 | 10.181 | 10.187 | 9.893 | 10.025 | 10.044 |
| LOD | >0.13 | >0.008 | >0.009 | >0.03 | >0.10 | >0.03 | >0.07 | >0.008 | >0.10 | >1.0 | >0.009 | >0.009 |

Table T2S. Shows value of Hg analysis in leachate samples from medical masks.

| **Sample Identity** | **Hg** |
|---|---|
| | **ppb** |
| QC 0.5 | 0.5437 |
| Blank | <0.050 |
| Geji R2 | <0.050 |
| Duronic R2 | <0.050 |
| Baltic R2 | <0.050 |
| Omnitex R2 | <0.050 |
| Soyes R2 | <0.050 |
| NHS | <0.050 |
| QC 0.5 | 0.5438 |
| LOD | >0.05 |

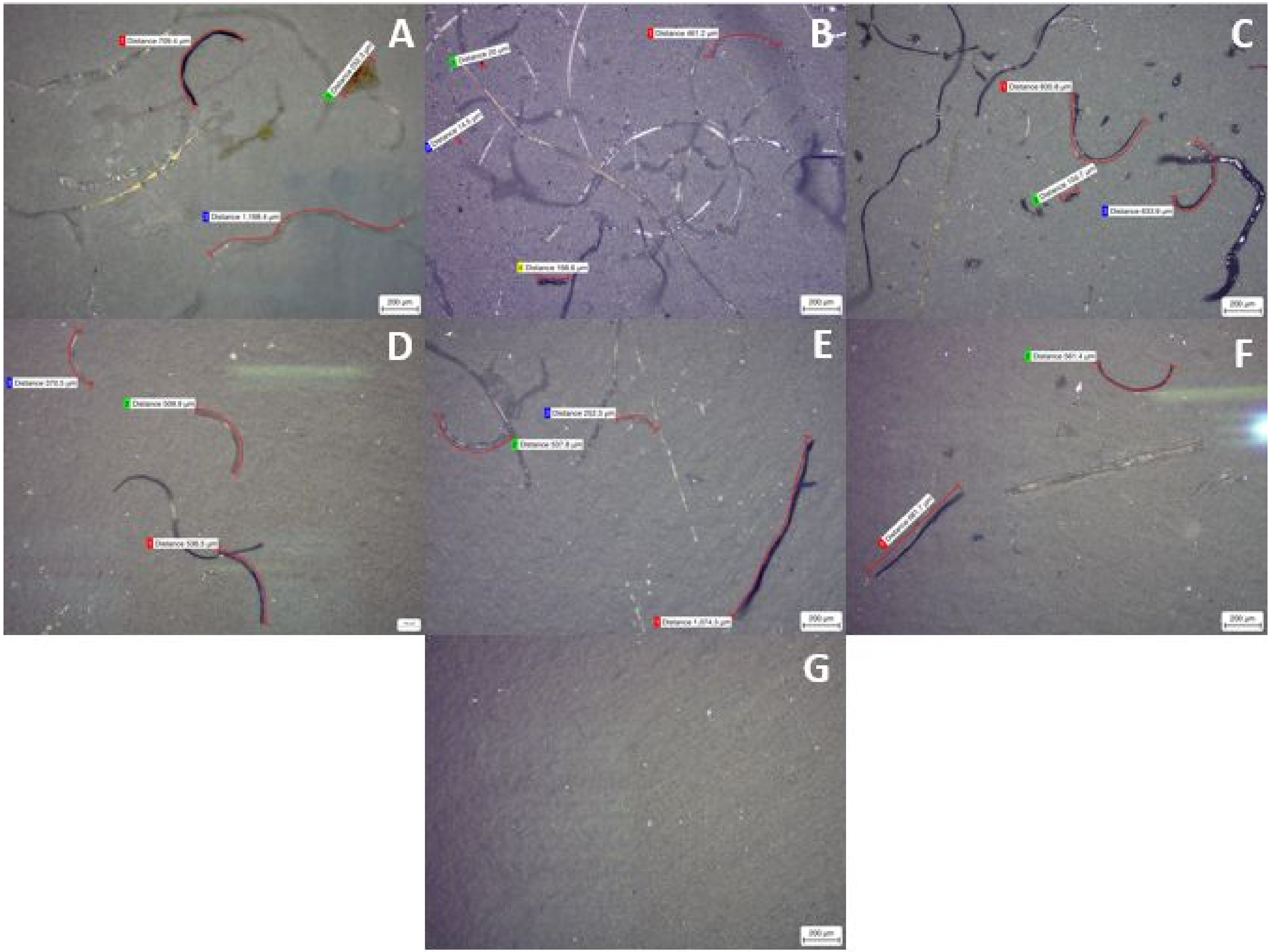


Figure 1S: Light microscope images at 50x overall magnification of 0.1 µm pore size membranes for all brands after filtration. (A) Baltic, (B) Geji, (C) Soyes (all FFP2), (D) Duronic, (E) NHS, (F) Omnitex (all Type IIR) and (G) Blank.

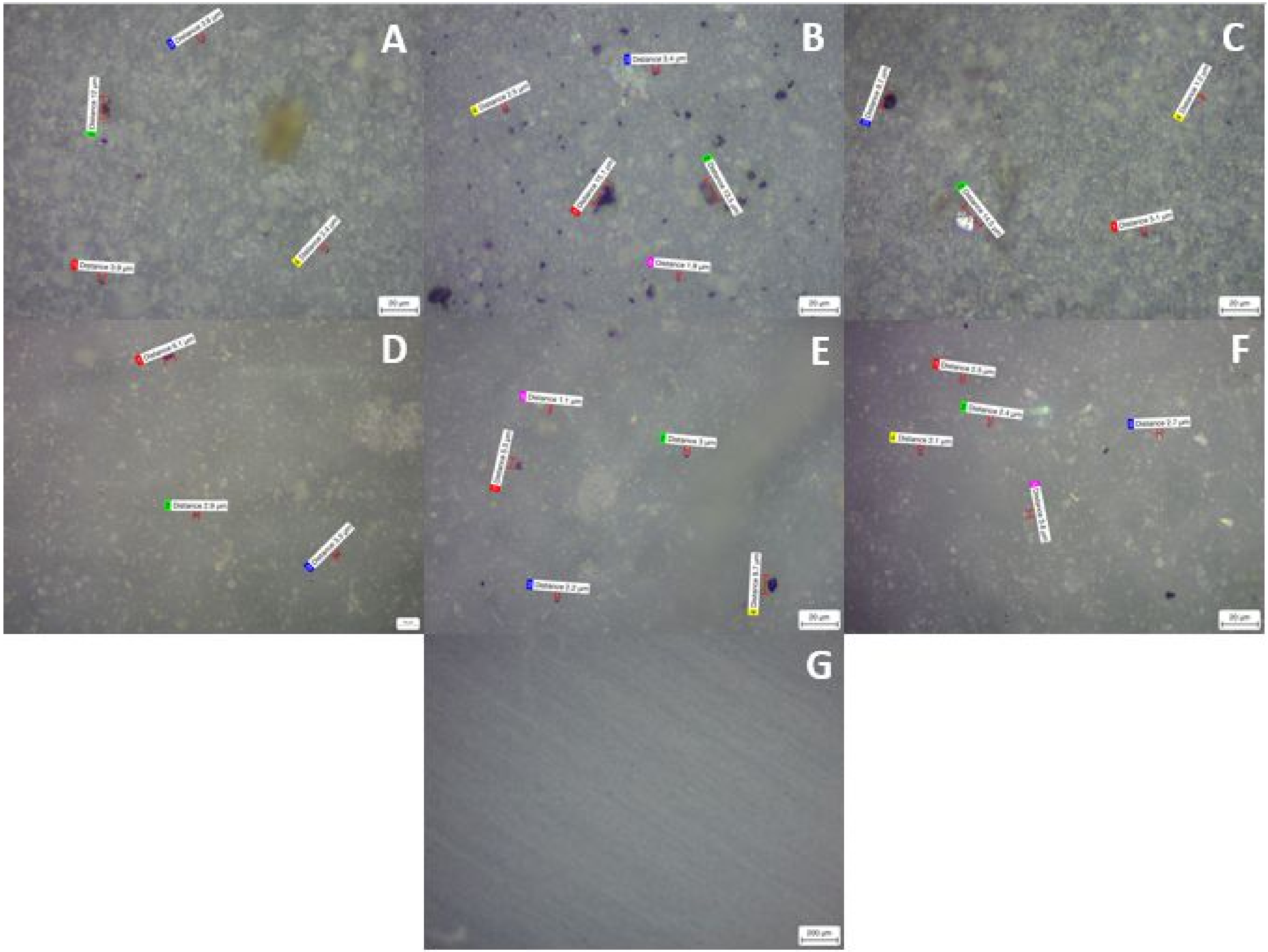


Figure 2S: Light microscope images at 500x overall magnification of 0.1 µm pore size membranes for all brands after filtration. (A) Baltic, (B) Geji, (C) Soyes (all FFP2), (D) Duronic, (E) NHS, (F) Omnitex (all Type IIR) and (G) Blank.

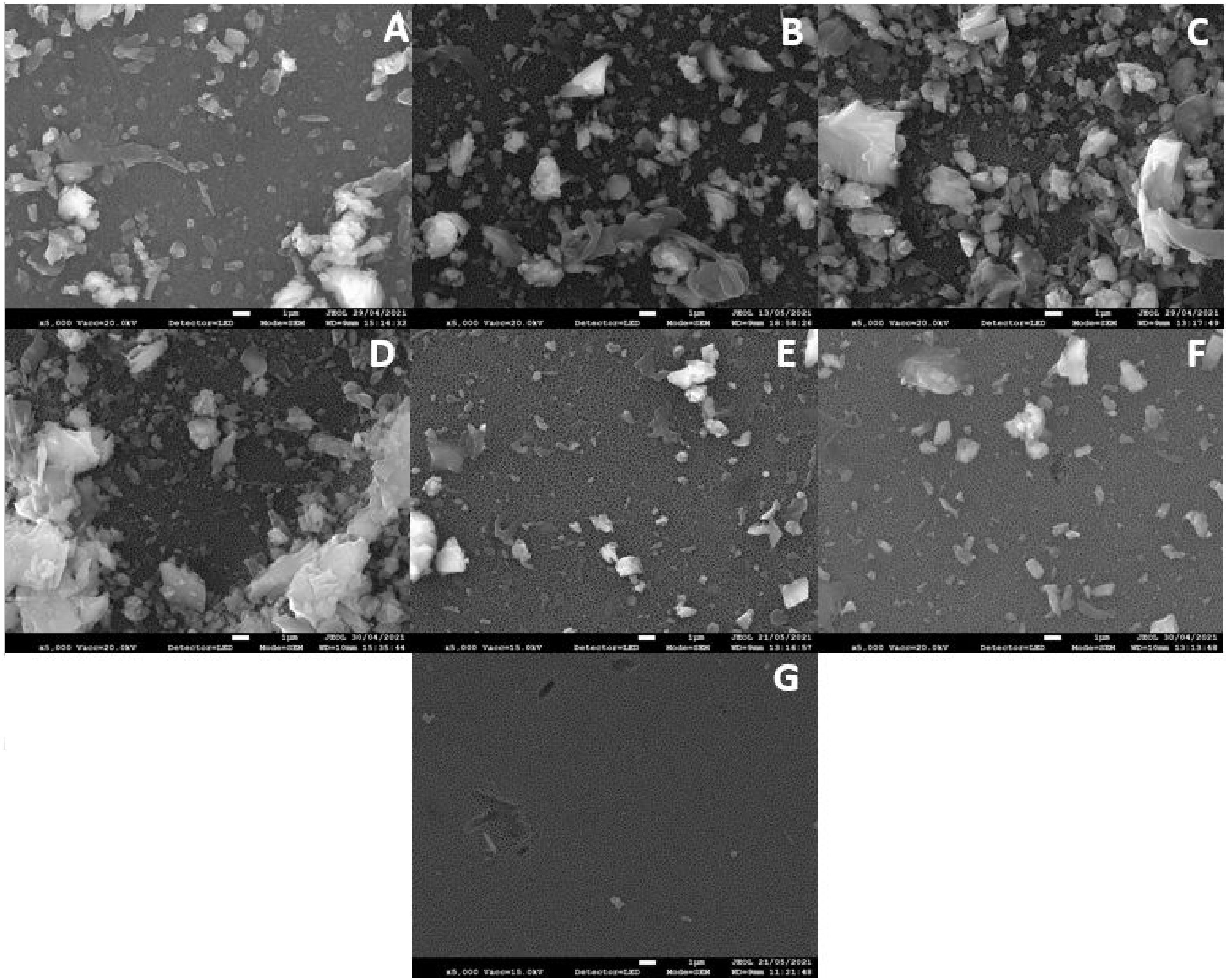


Figure 3S. FEG-SEM images of 0.1 µm pore size membranes for all brands at x5000. (A) Baltic, (B) Geji, (C) Soyes (all FFP2), (D) Duronic, (E) NHS, (F) Omnitex (all Type IIR) and (G) Blank.

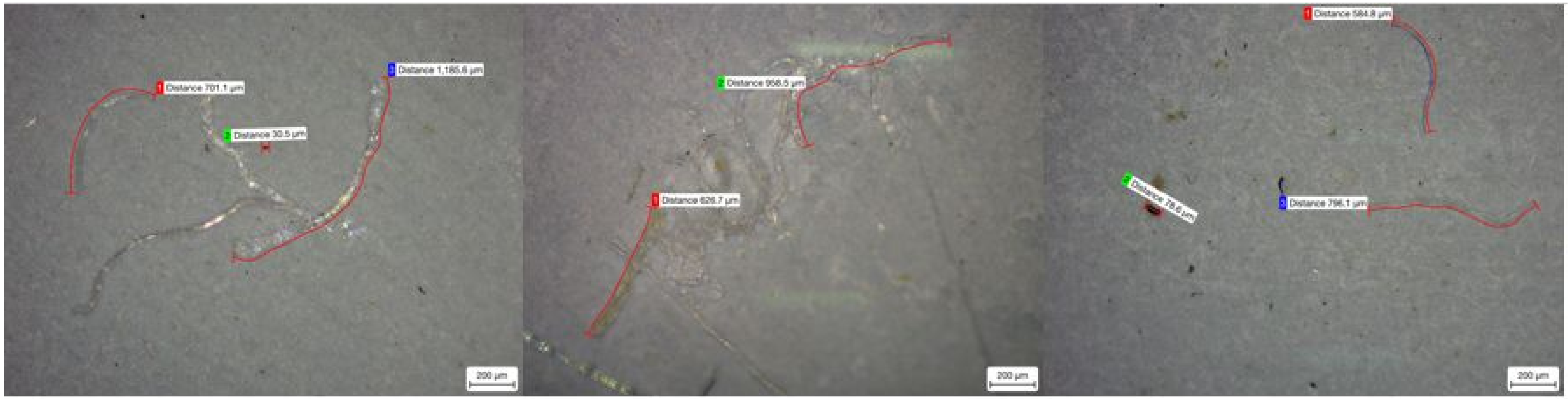


Figure 4S. Some replicates for light microscope images, at 50x overall magnification, of 0.02 µm pore size membranes. Baltic brand.

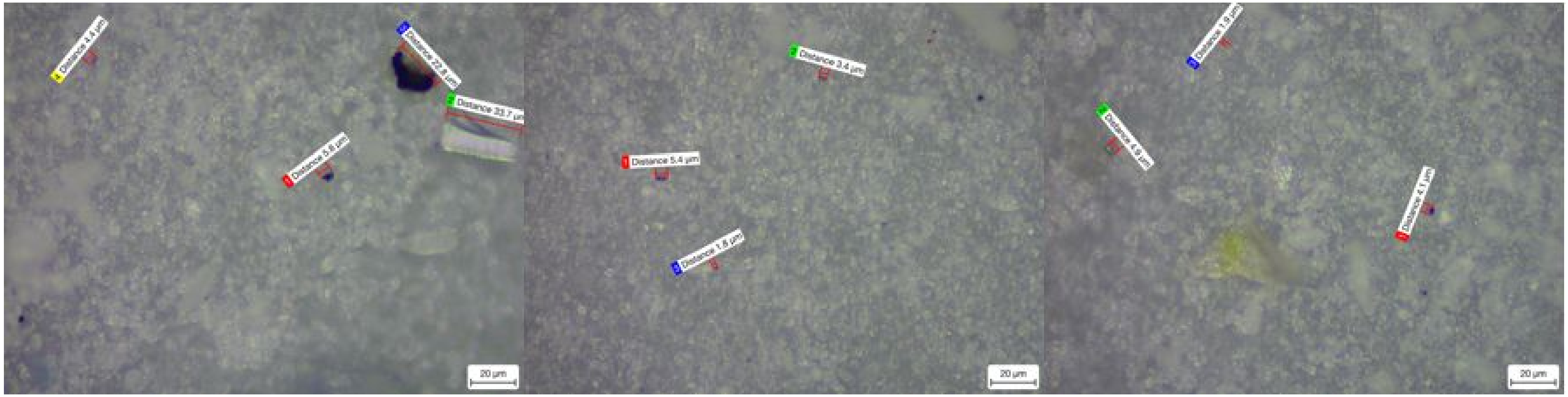


Figure 5S. Some replicates for light microscope images, at 500x overall magnification, of 0.02 µm pore size membranes. Baltic brand.

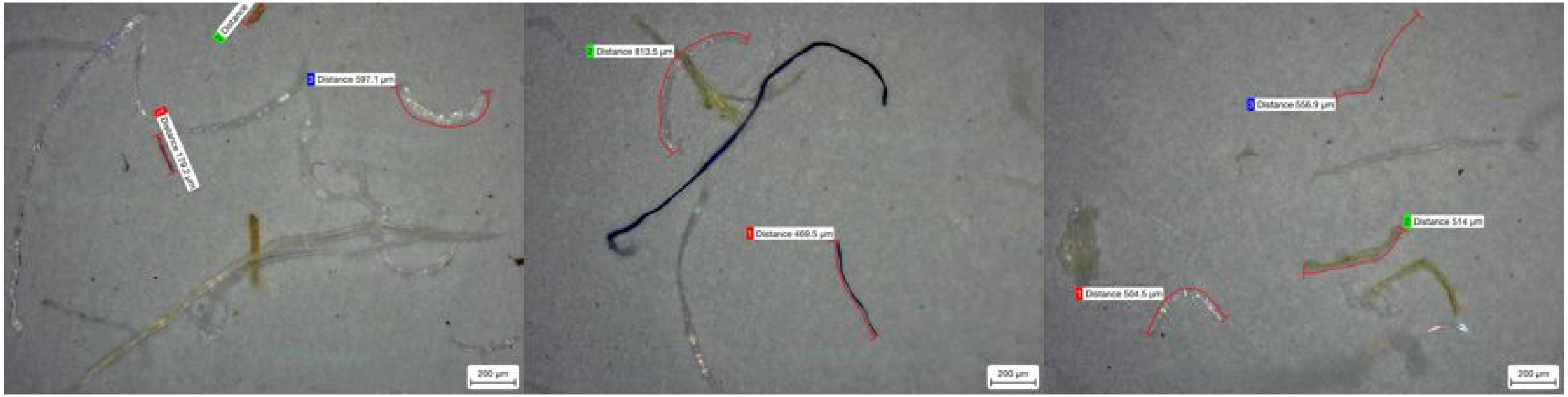

Figure 6S. Some replicates for light microscope images, at 50x overall magnification, of 0.1 µm pore size membranes. Baltic brand.

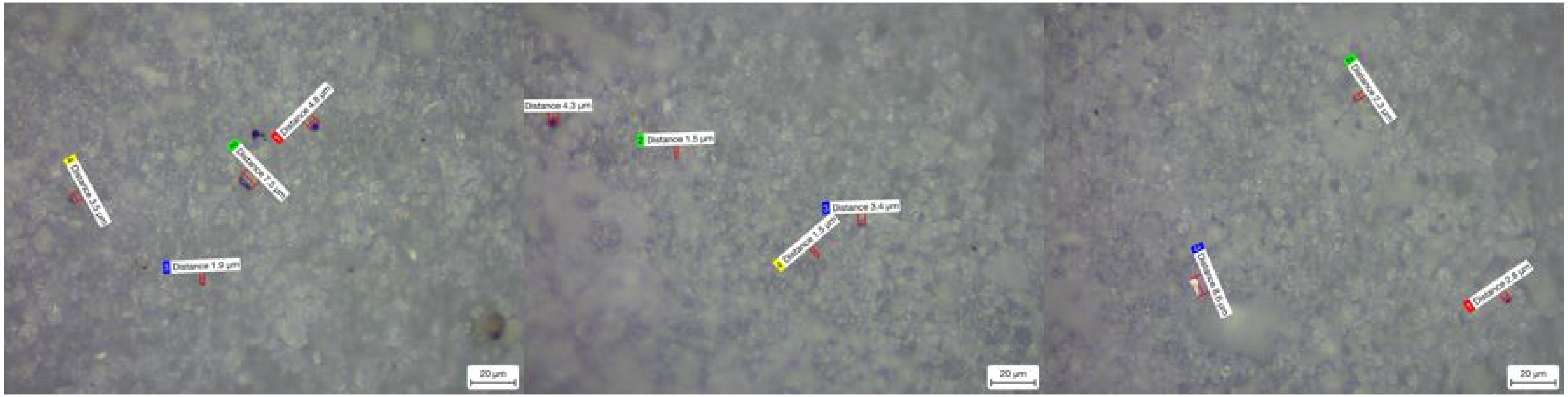

Figure 7S. Some replicates for light microscope images, at 500x overall magnification, of 0.1 µm pore size membranes. Baltic brand.

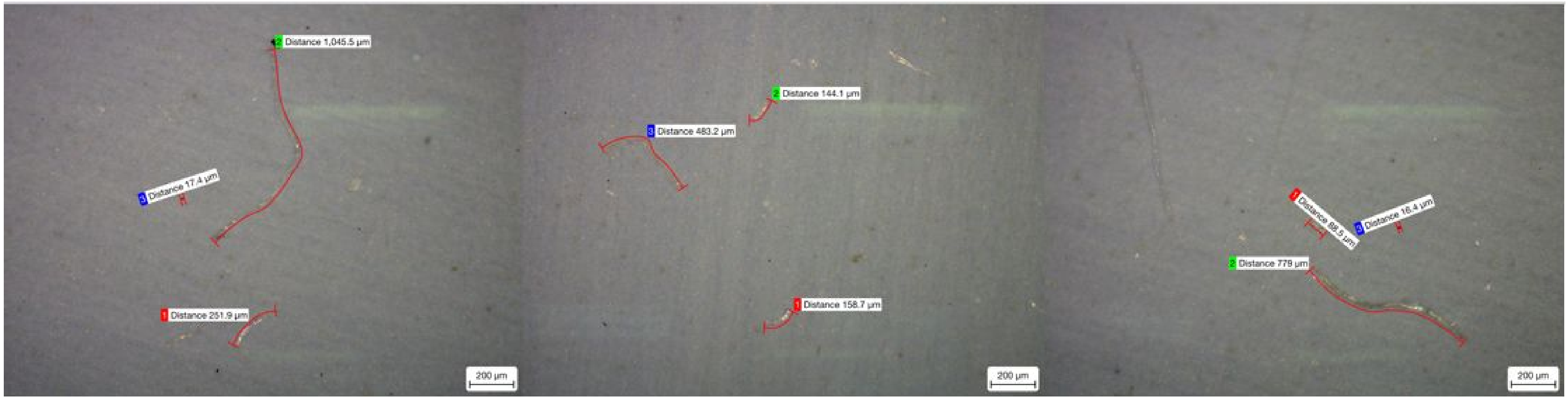


Figure 8S. Some replicates for light microscope images, at 50x overall magnification, of 0.02 µm pore size membranes. Duronic brand.

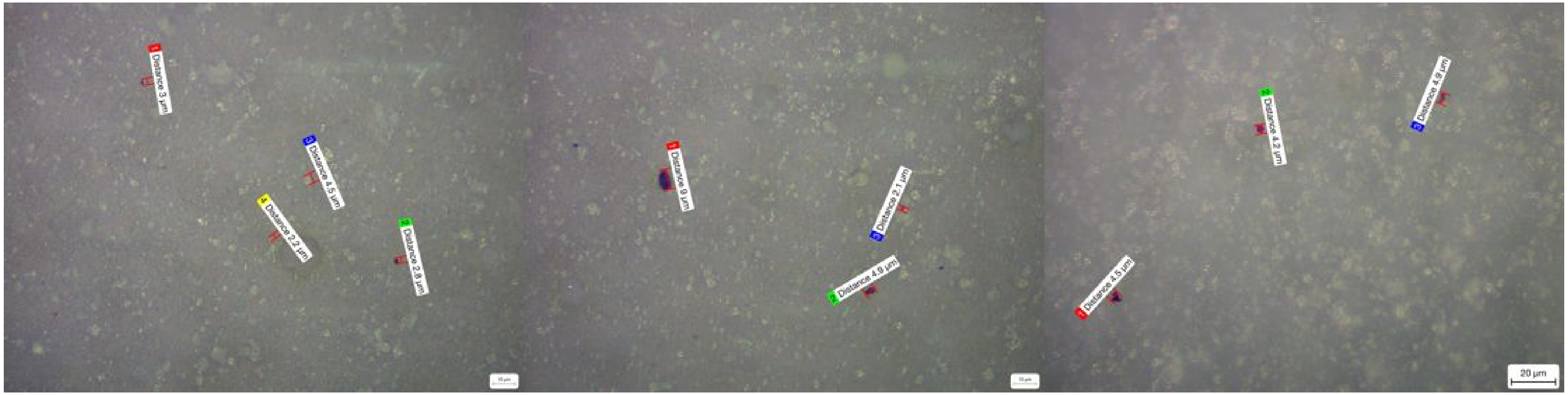


Figure 9S. Some replicates for light microscope images, at 500x overall magnification, of 0.02 µm pore size membranes. Duronic brand.

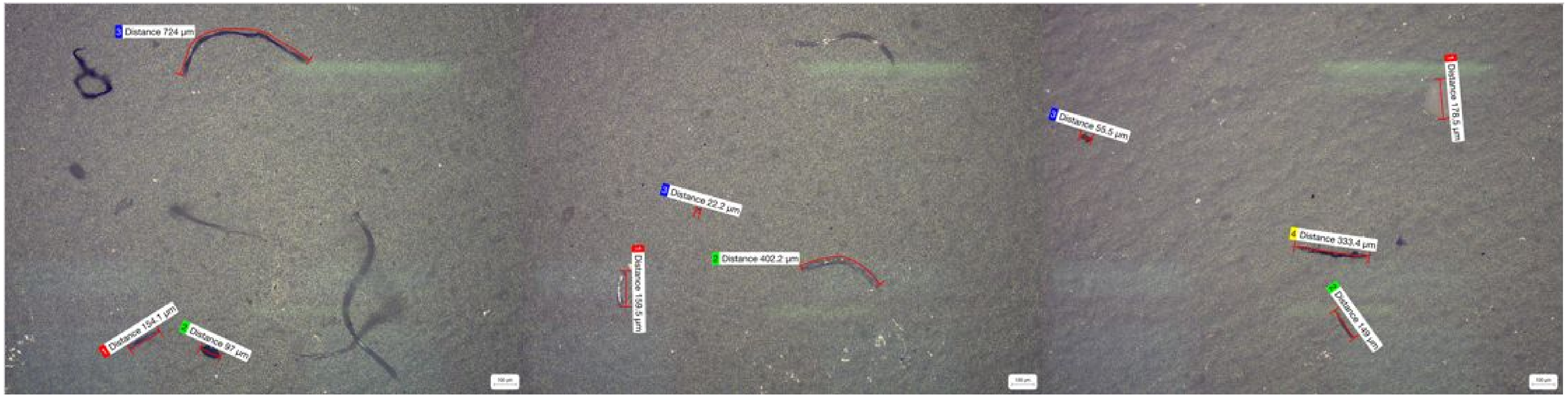


Figure 10S. Some replicates for light microscope images, at 50x overall magnification, of 0.1 µm pore size membranes. Duronic brand.

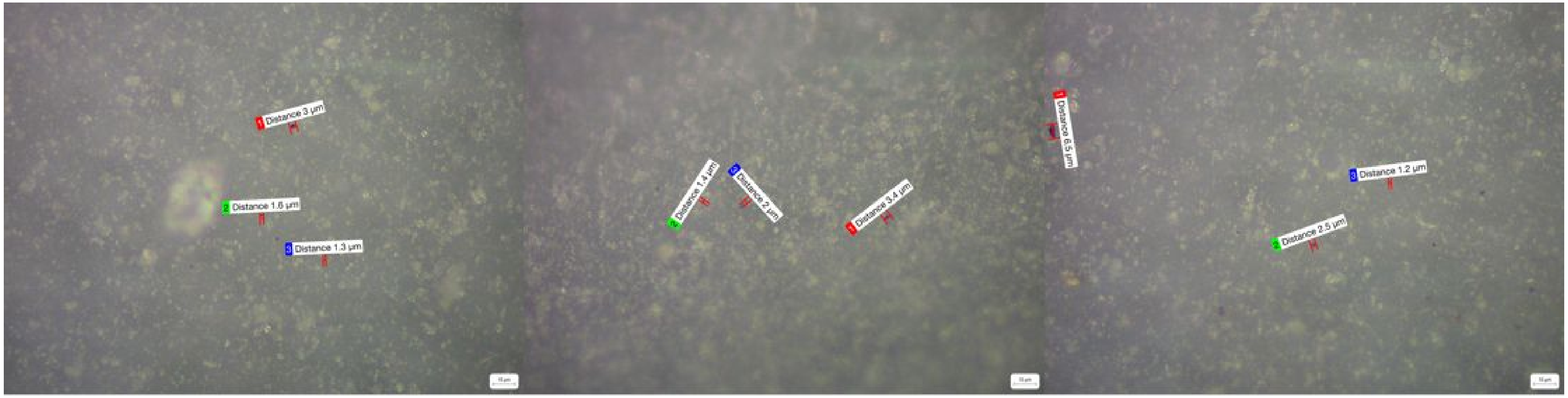


Figure 11S. Some replicates for light microscope images, at 500x overall magnification, of 0.1 µm pore size membranes. Duronic brand.

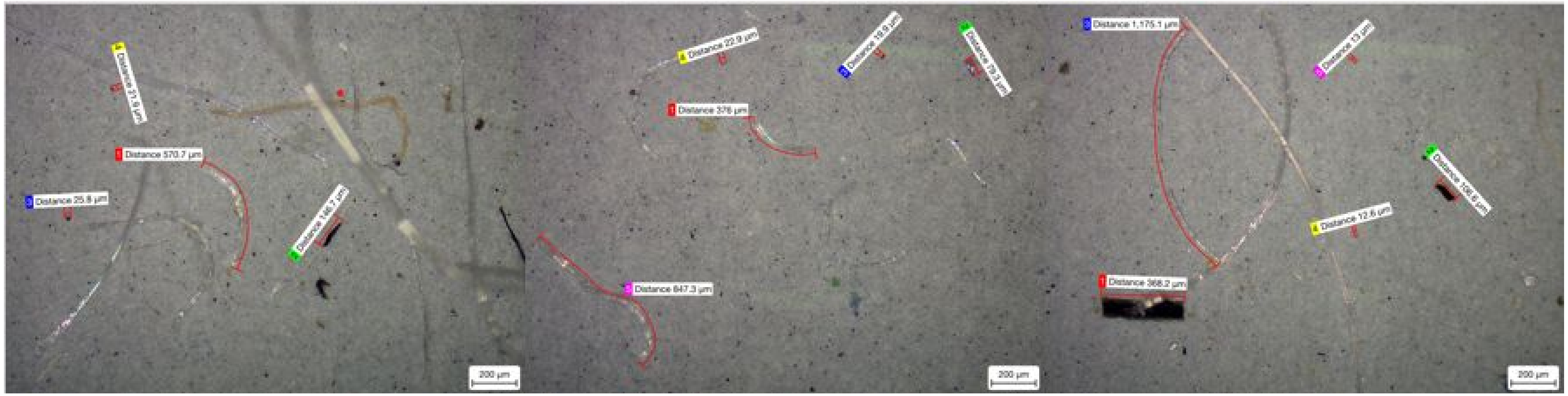

Figure 12S. Some replicates for light microscope images, at 50x overall magnification, of 0.02 µm pore size membranes. Geji brand.

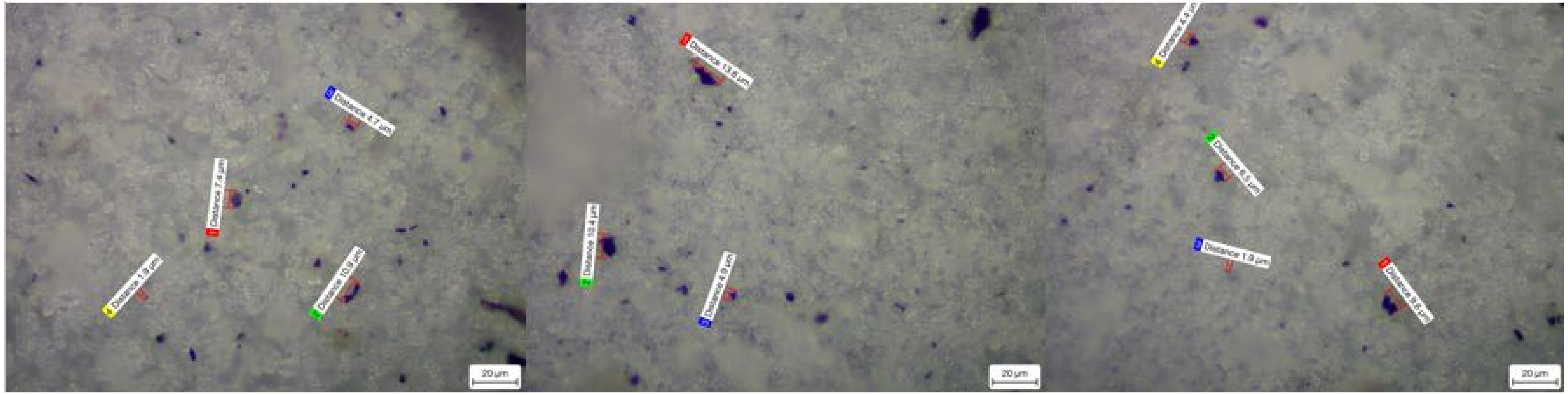

Figure 13S. Some replicates for light microscope images, at 500x overall magnification, of 0.02 µm pore size membranes. Geji brand.

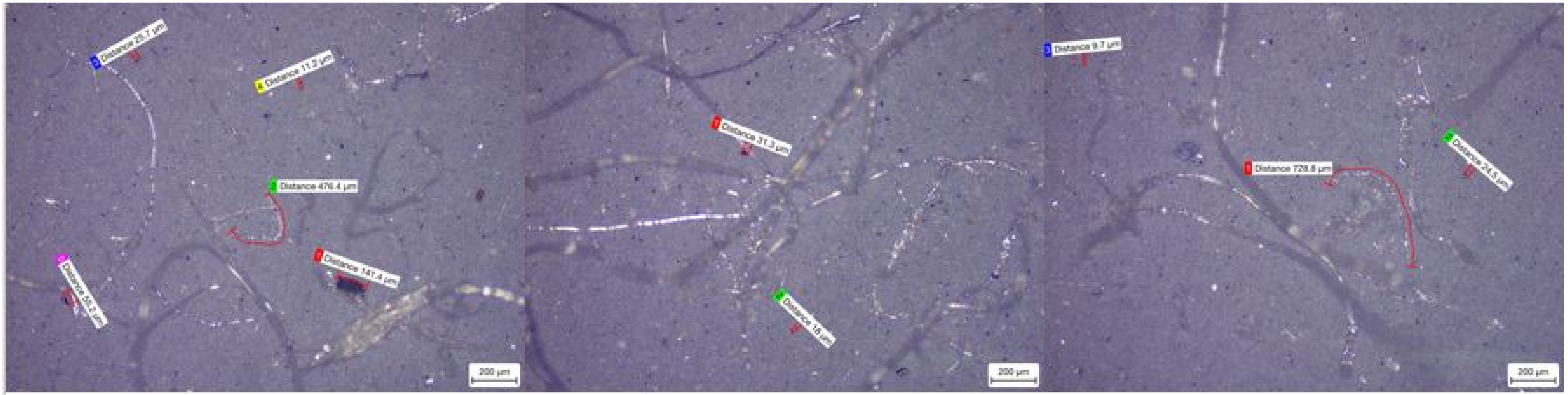

Figure 14S. Some replicates for light microscope images, at 50x overall magnification, of 0.1 µm pore size membranes. Geji brand.

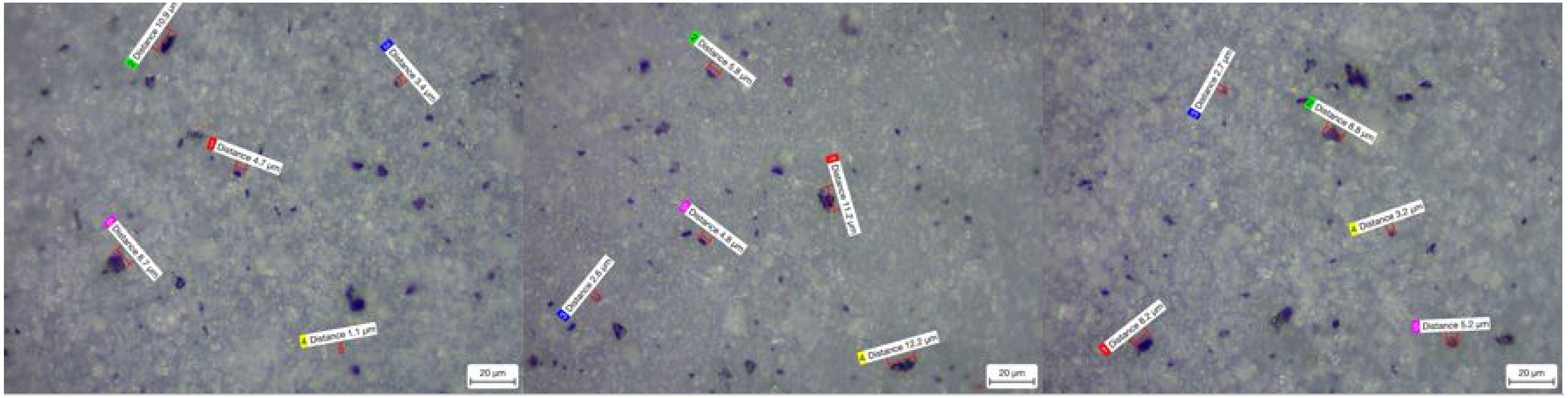

Figure 15S. Some replicates for light microscope images, at 500x overall magnification, of 0.1 µm pore size membranes. Geji brand.

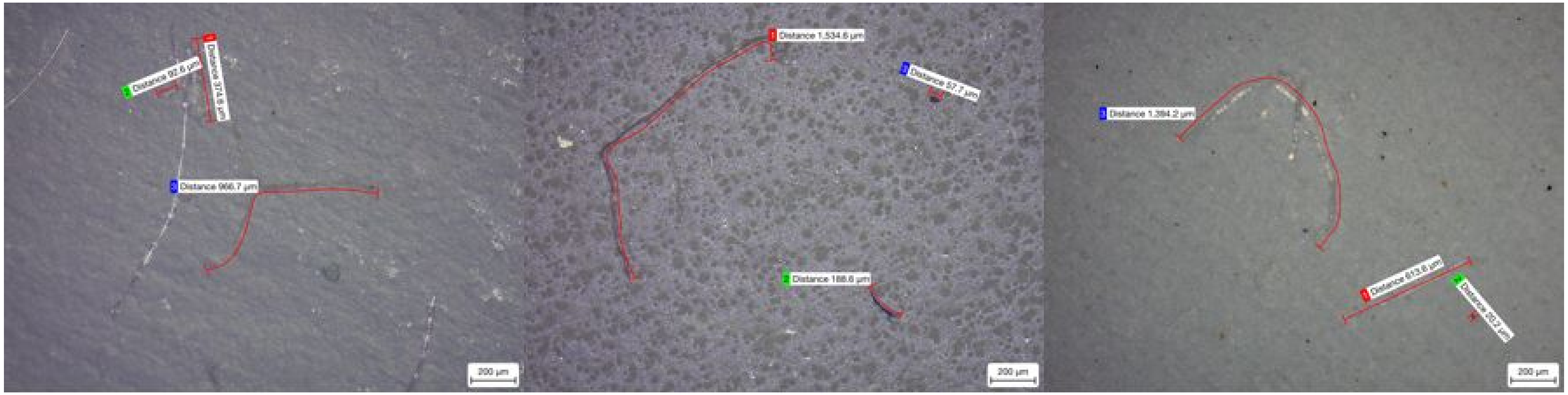


Figure 16S. Some replicates for light microscope images, at 50x overall magnification, of 0.02 µm pore size membranes. NHS brand.

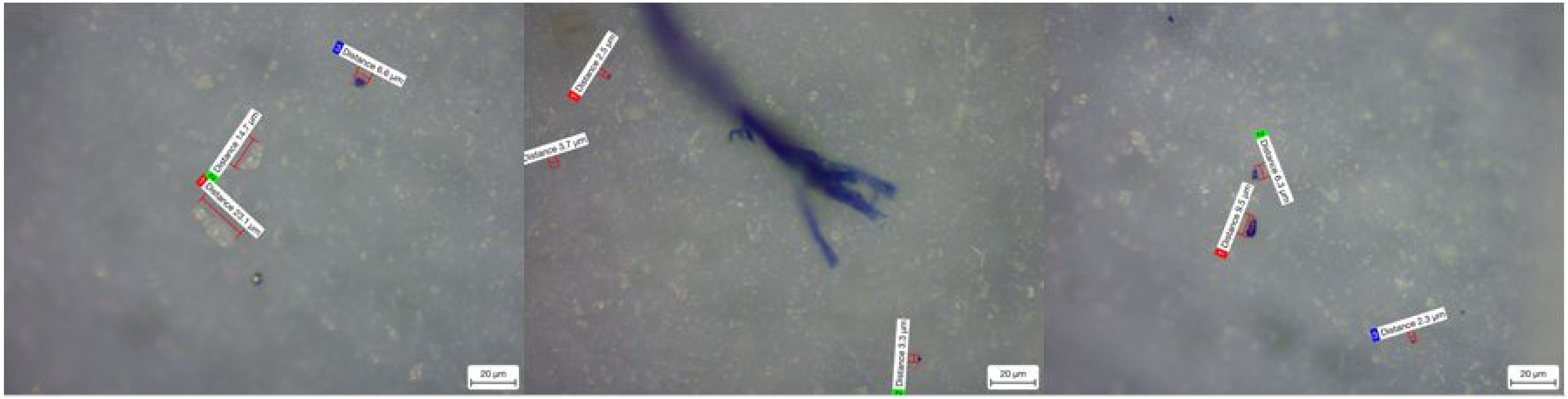


Figure 17S. Some replicates for light microscope images, at 500x overall magnification, of 0.02 µm pore size membranes. NHS brand.

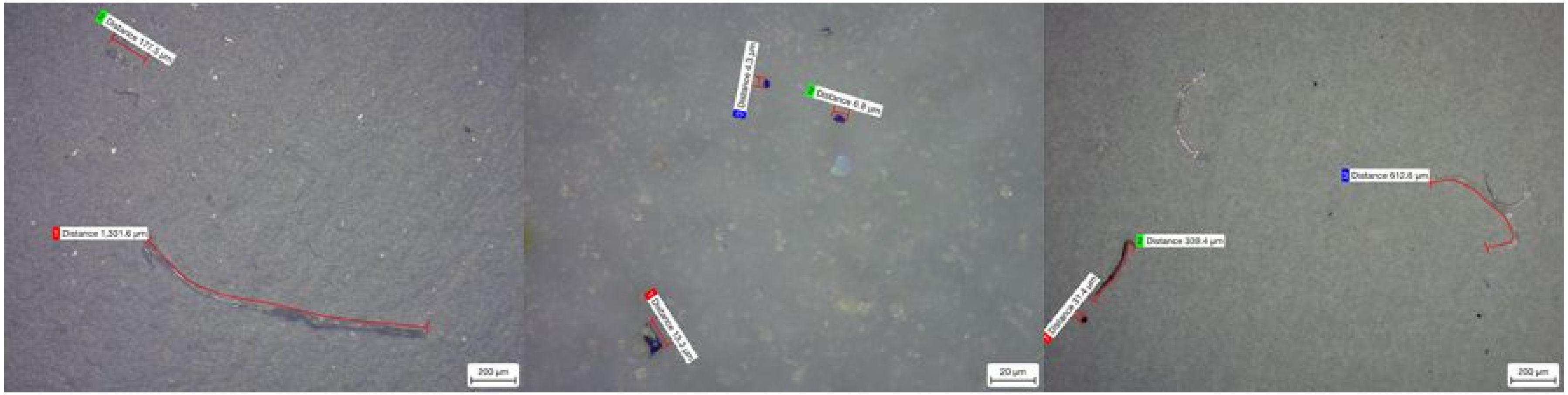


Figure 18S. Some replicates for light microscope images, at 50x overall magnification, of 0.1 µm pore size membranes. NHS brand.

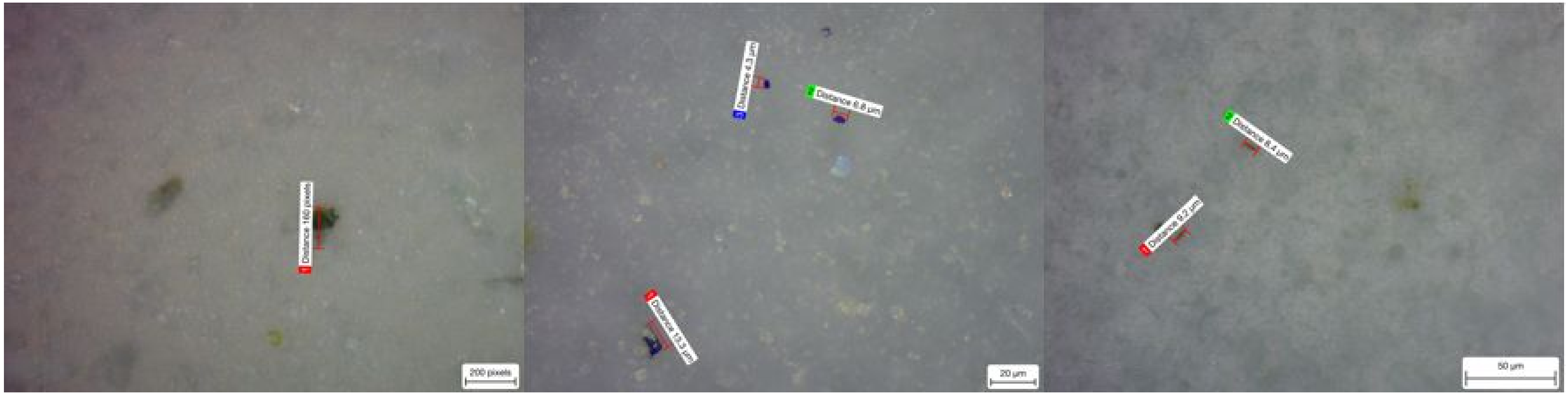


Figure 19S. Some replicates for light microscope images, at 500x overall magnification, of 0.1 µm pore size membranes. NHS brand.

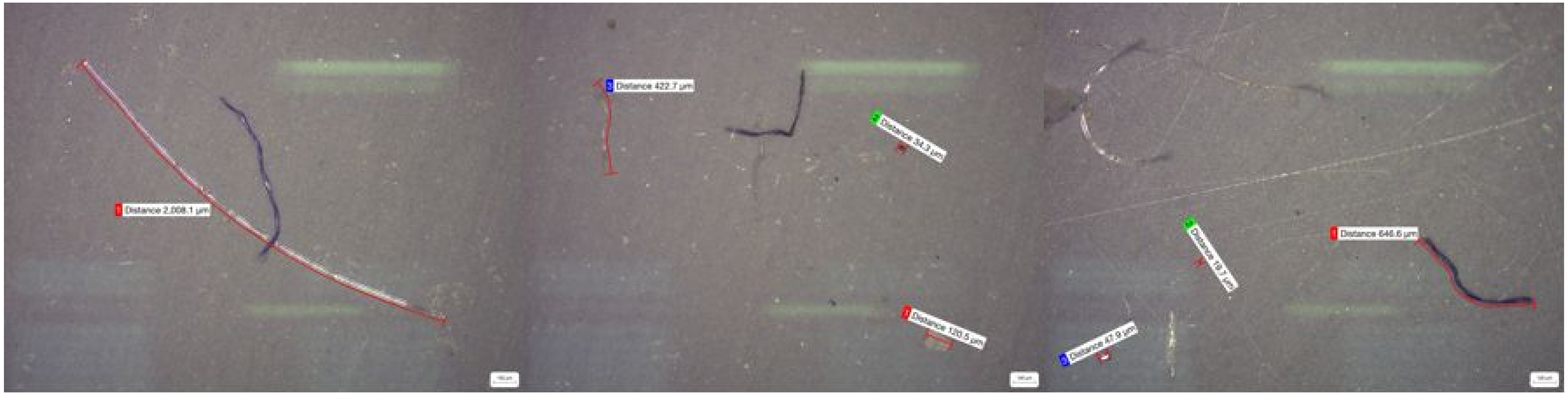


Figure 20S. Some replicates for light microscope images, at 50x overall magnification, of 0.02 µm pore size membranes. Omnitex brand.

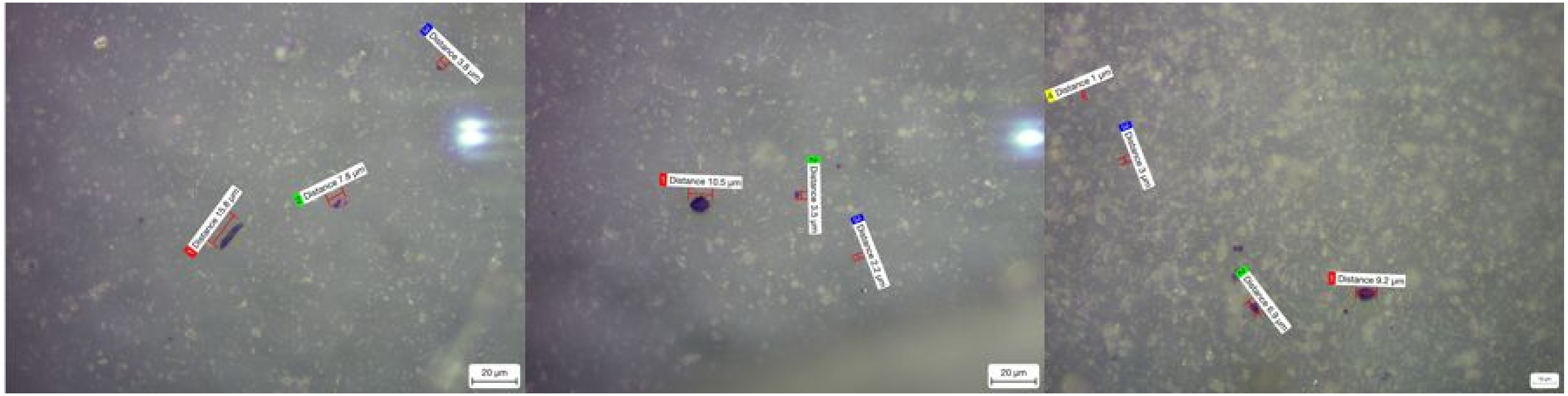


Figure 21S. Some replicates for light microscope images, at 500x overall magnification, of 0.02 µm pore size membranes. Omnitex brand.

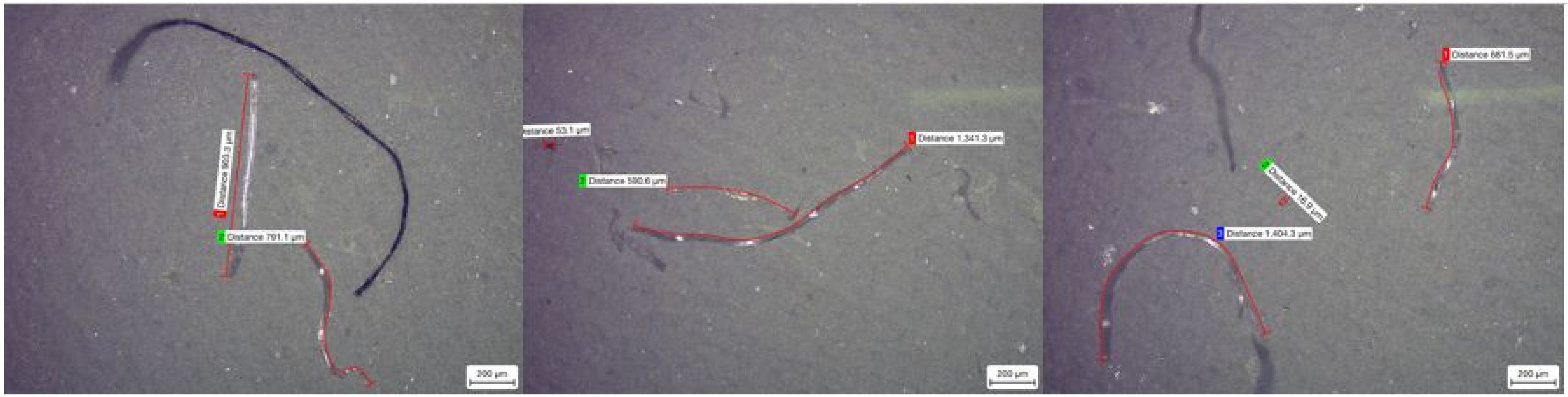


Figure 22S. Some replicates for light microscope images, at 50x overall magnification, of 0.1 µm pore size membranes. Omnitex brand.

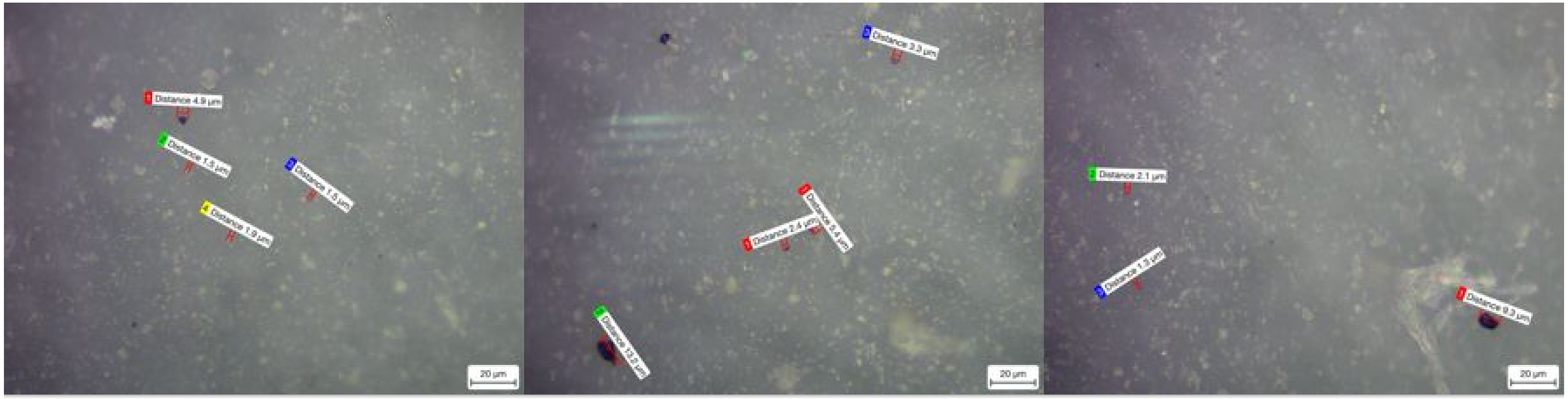


Figure 23S. Some replicates for light microscope images, at 500x overall magnification, of 0.1 µm pore size membranes. Omnitex brand.

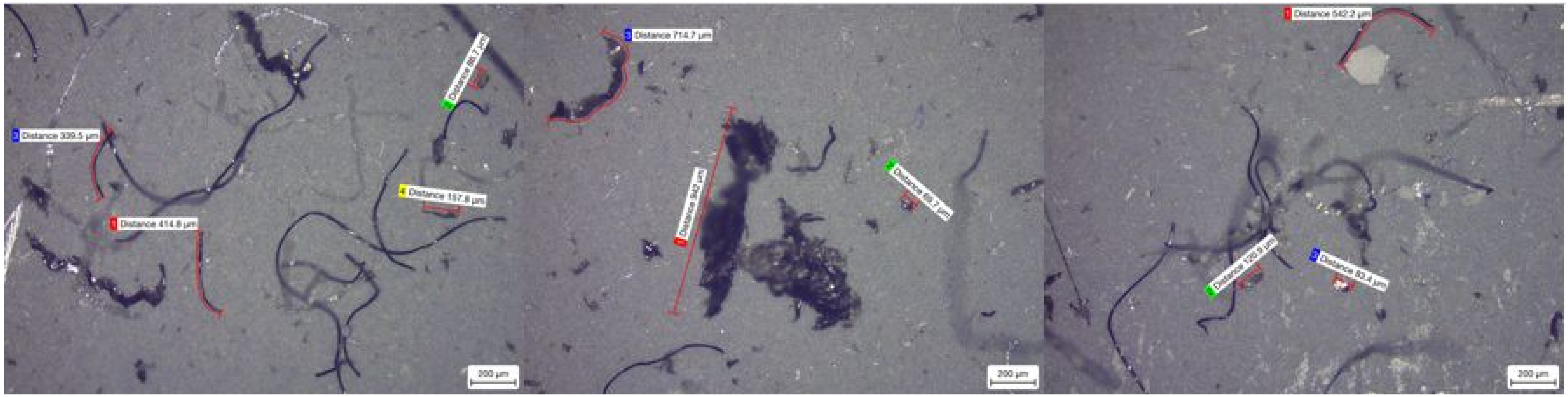


Figure 24S. Some replicates for light microscope images, at 50x overall magnification, of 0.02 µm pore size membranes. Soyes brand.

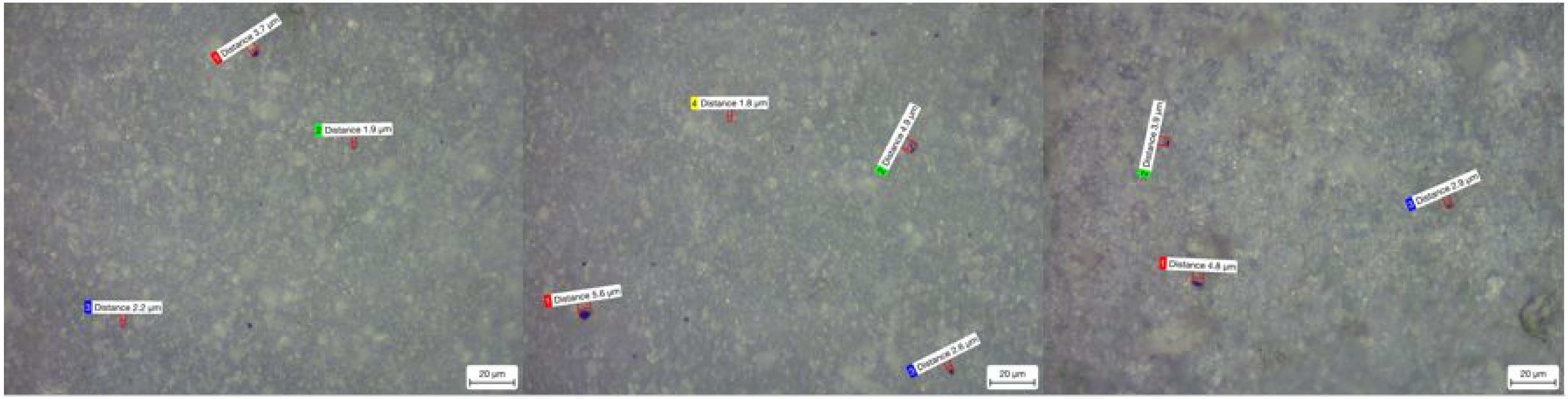


Figure 25S. Some replicates for light microscope images, at 500x overall magnification, of 0.02 µm pore size membranes. Soyes brand.

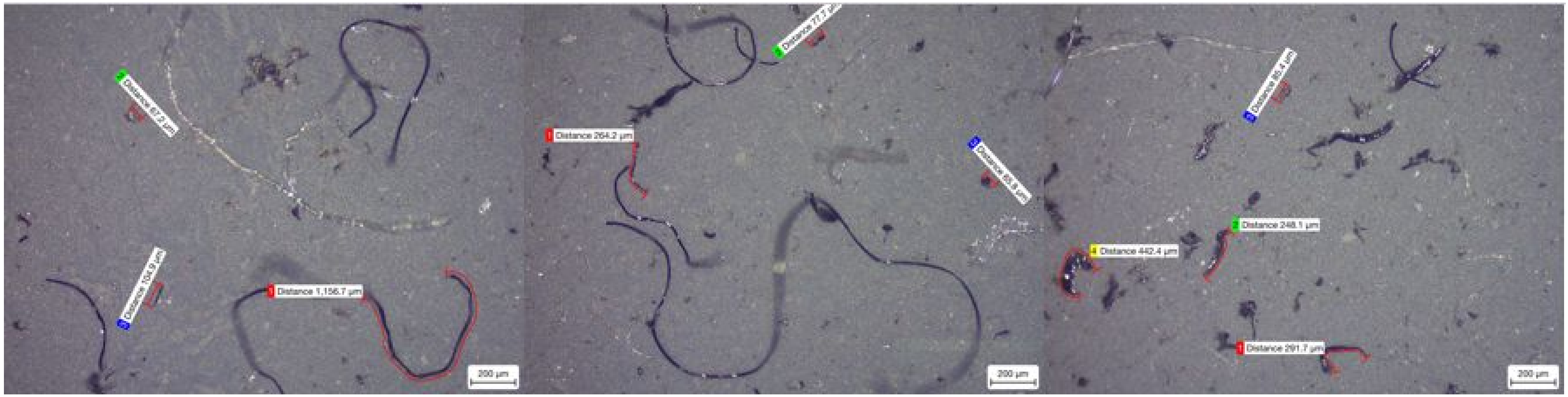


Figure 26S. Some replicates for light microscope images, at 50x overall magnification, of 0.1 µm pore size membranes. Soyes brand.

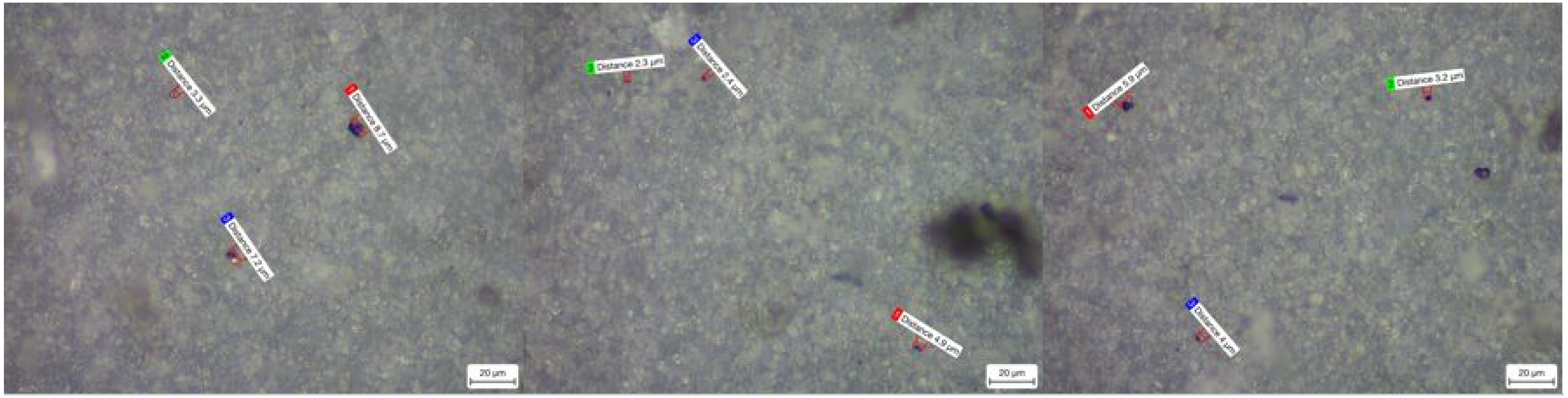


Figure 27S. Some replicates for light microscope images, at 500x overall magnification, of 0.1 µm pore size membranes. Soyes brand.

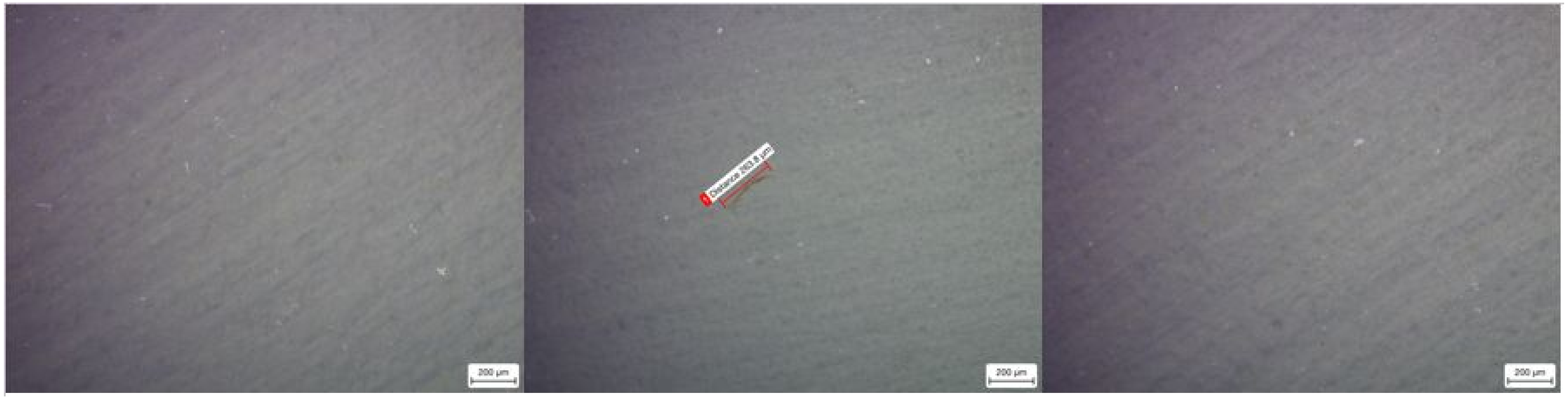


Figure 28S. Some replicates for light microscope images, at 50x overall magnification, of 0.02 µm pore size membranes. Blank.

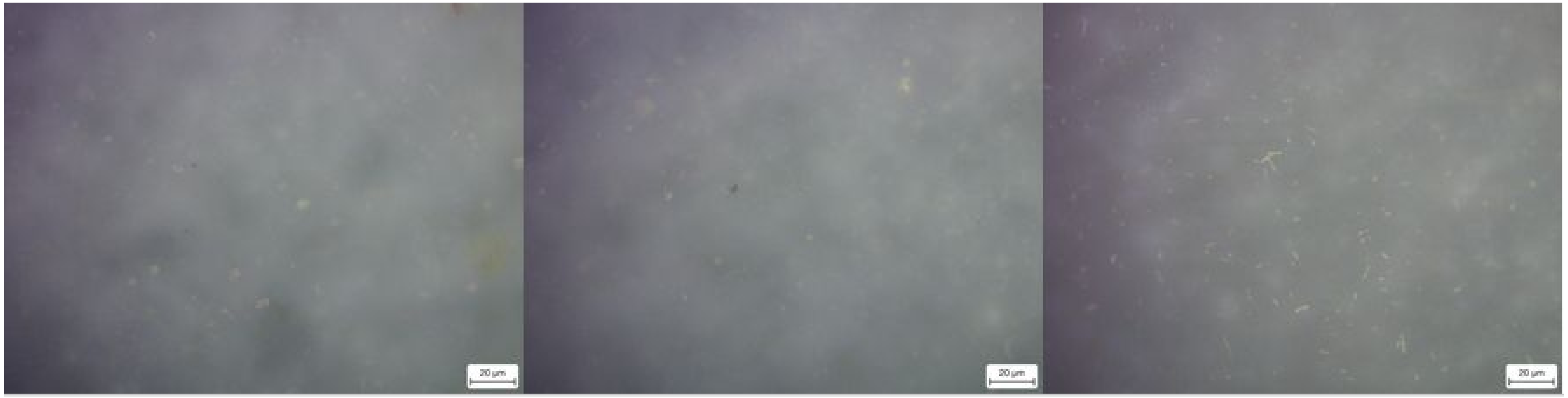


Figure 29S. Some replicates for light microscope images, at 500x overall magnification, of 0.02 µm pore size membranes. Blank.

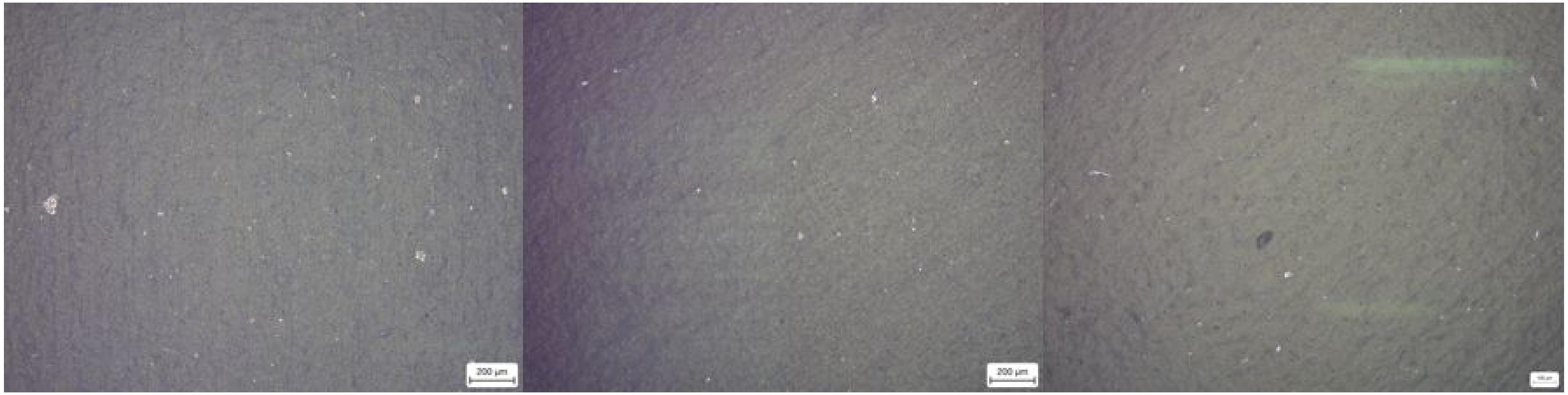


Figure 30S. Some replicates for light microscope images, at 50x overall magnification, of 0.1 µm pore size membranes. Blank.

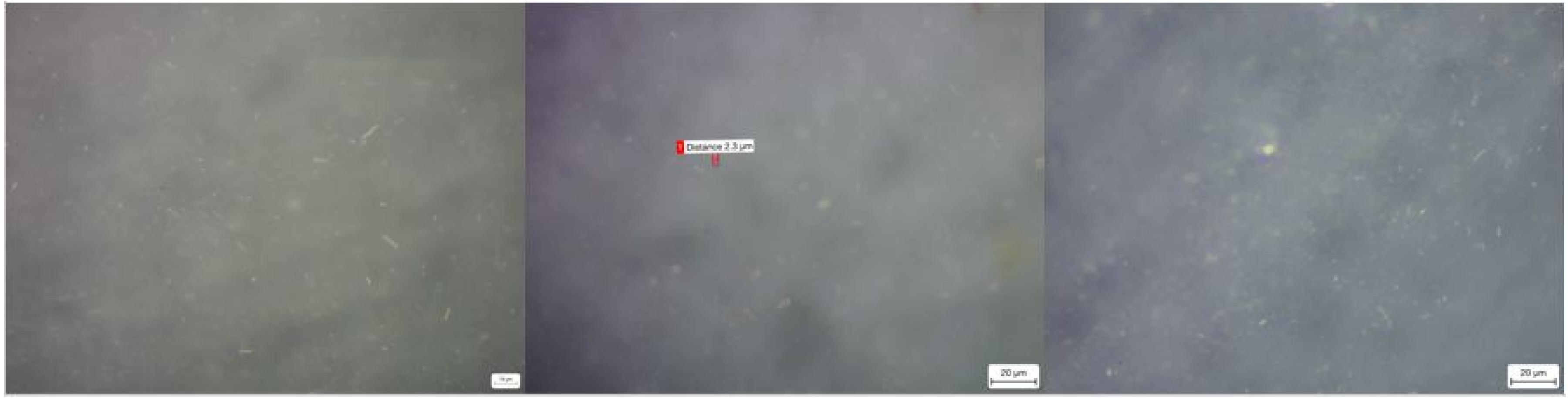


Figure 31S. Some replicates for light microscope images, at 500x overall magnification, of 0.1 µm pore size membranes. Blank.

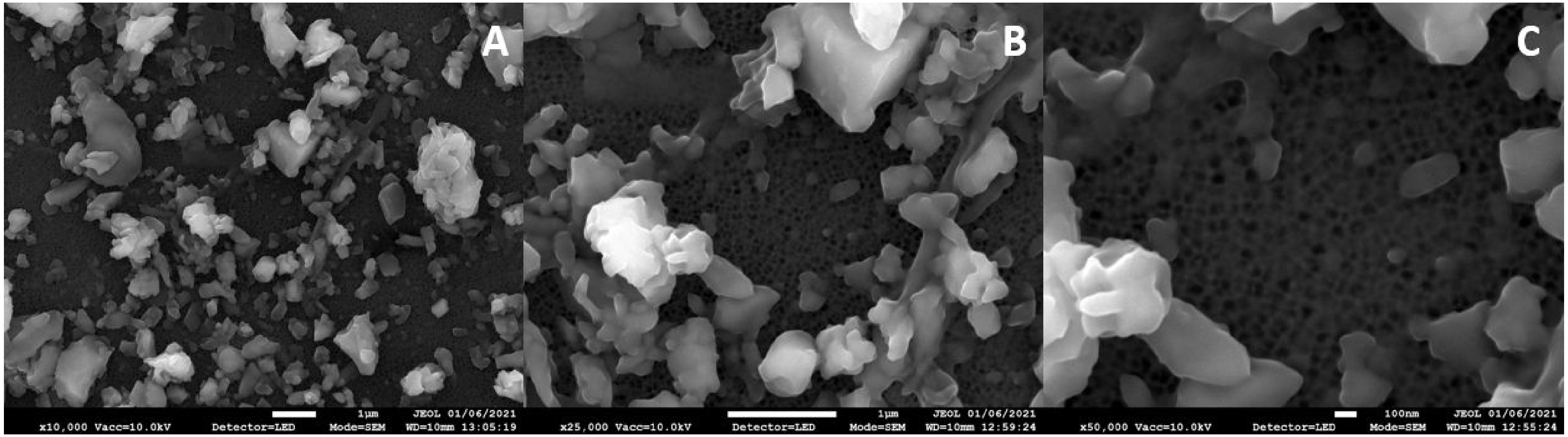


Figure 32S. FEG-SEM replicates of 0.02 µm pore size membranes for Brand Baltic (FFP2). (A) 10000x, (B) 25000x and (C) 50000x.

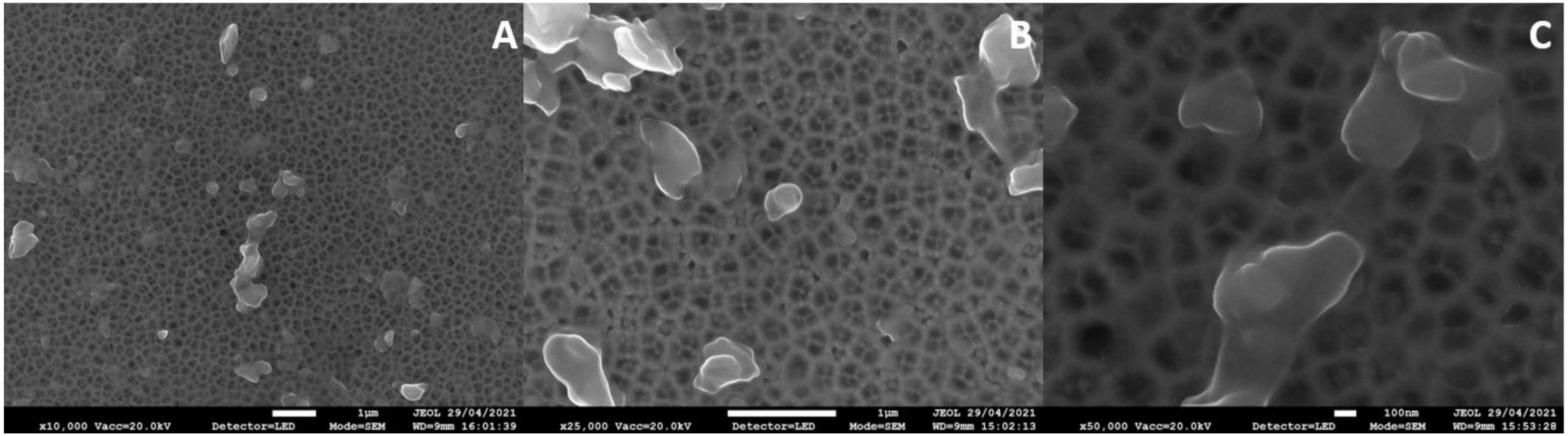


Figure 33S. FEG-SEM replicates of 0.1 µm pore size membranes for Brand Baltic (FFP2). (A) 10000x, (B) 25000x and (C) 50000x.

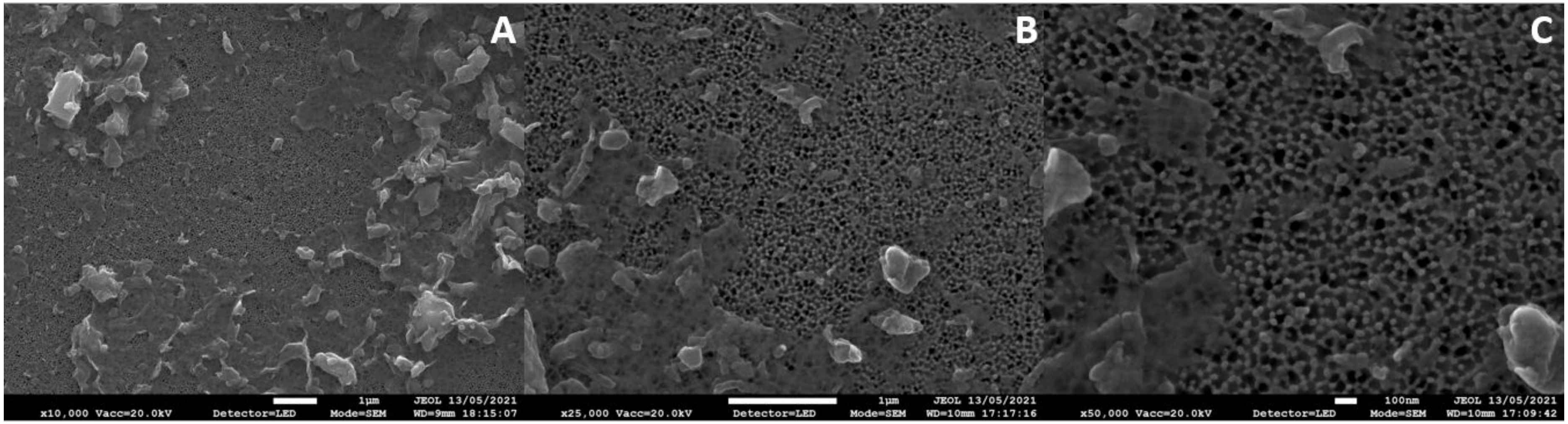


Figure 34S. FEG-SEM replicates of 0.02 µm pore size membranes for Brand Duronic (IIR). (A) 10000x, (B) 25000x and (C) 50000x.

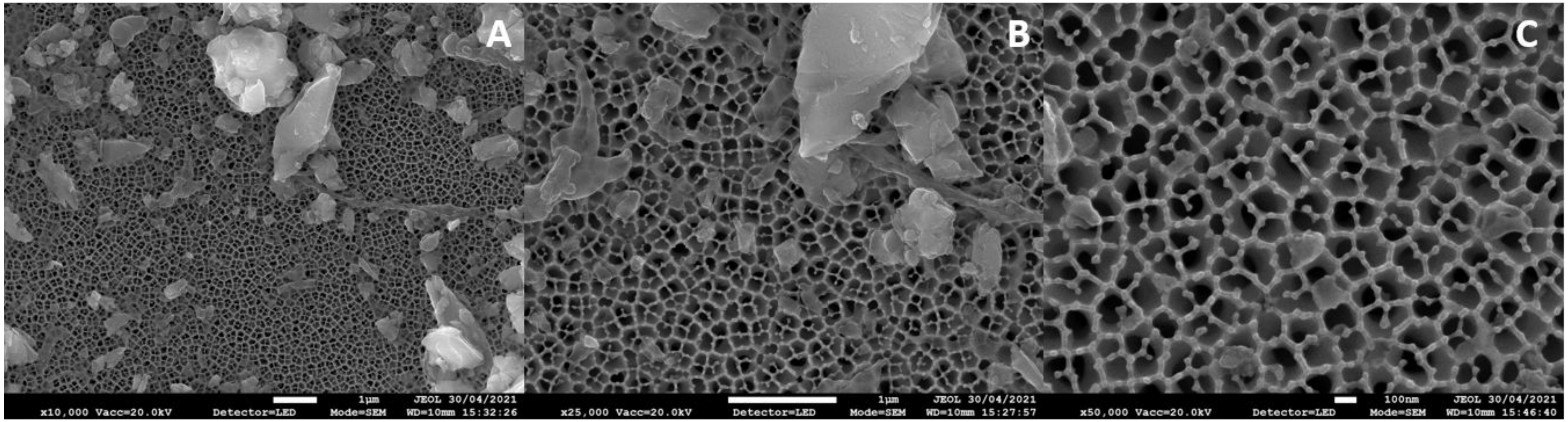


Figure 35S. FEG-SEM replicates of 0.1 µm pore size membranes for Brand Duronic (IIR). (A) 10000x, (B) 25000x and (C) 50000x.

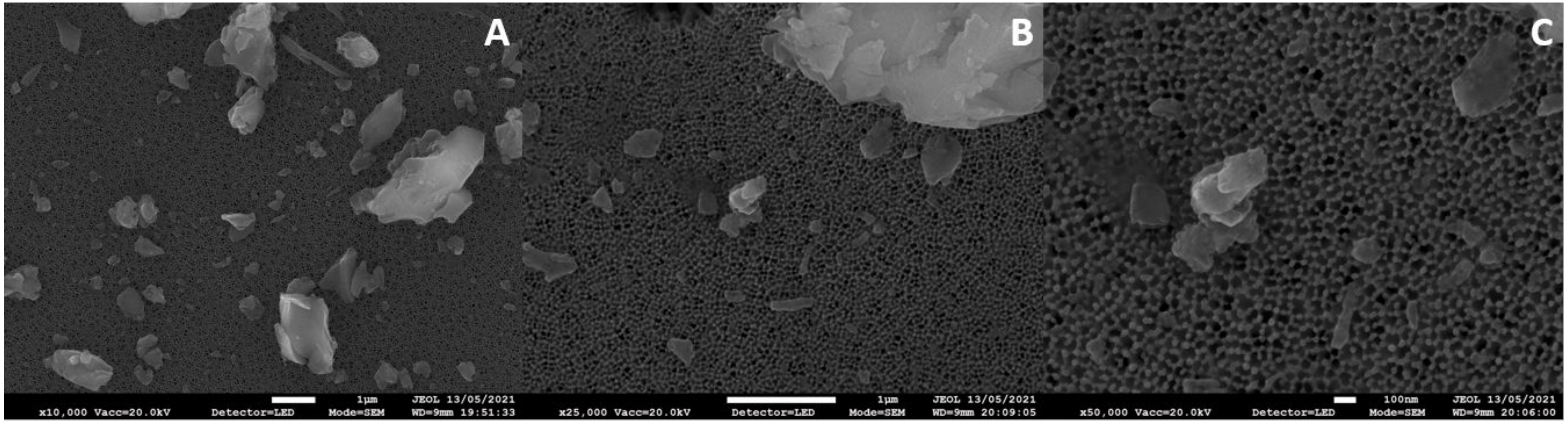


Figure 36S. FEG-SEM replicates of 0.02 µm pore size membranes for Brand Geji (FFP2). (A) 10000x, (B) 25000x and (C) 50000x.

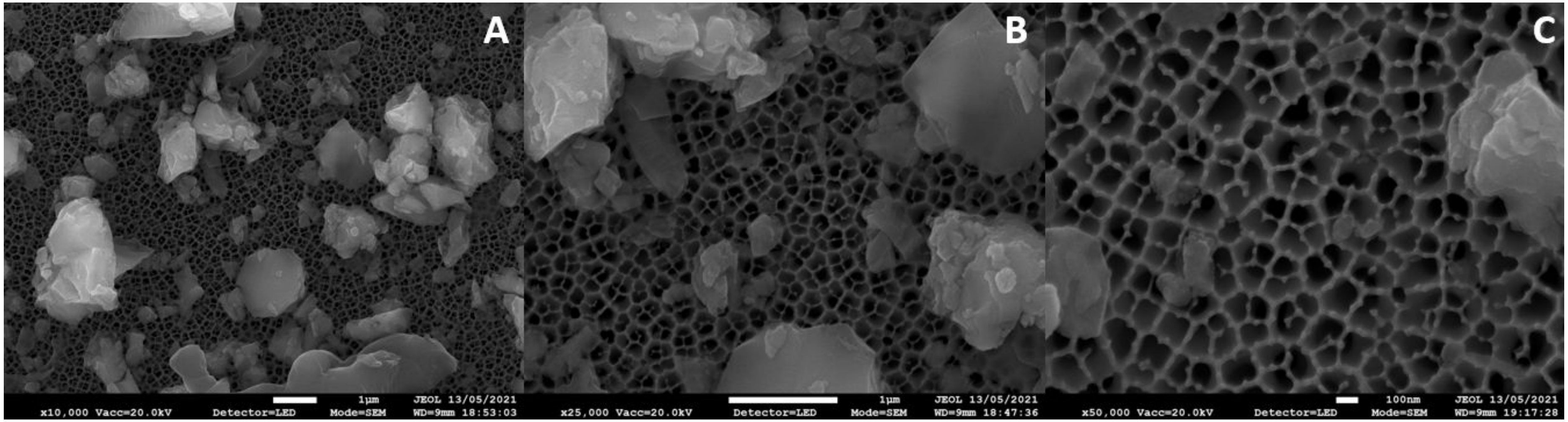


Figure 37S. FEG-SEM replicates of 0.1 µm pore size membranes for Brand Geji (FFP2). (A) 10000x, (B) 25000x and (C) 50000x.

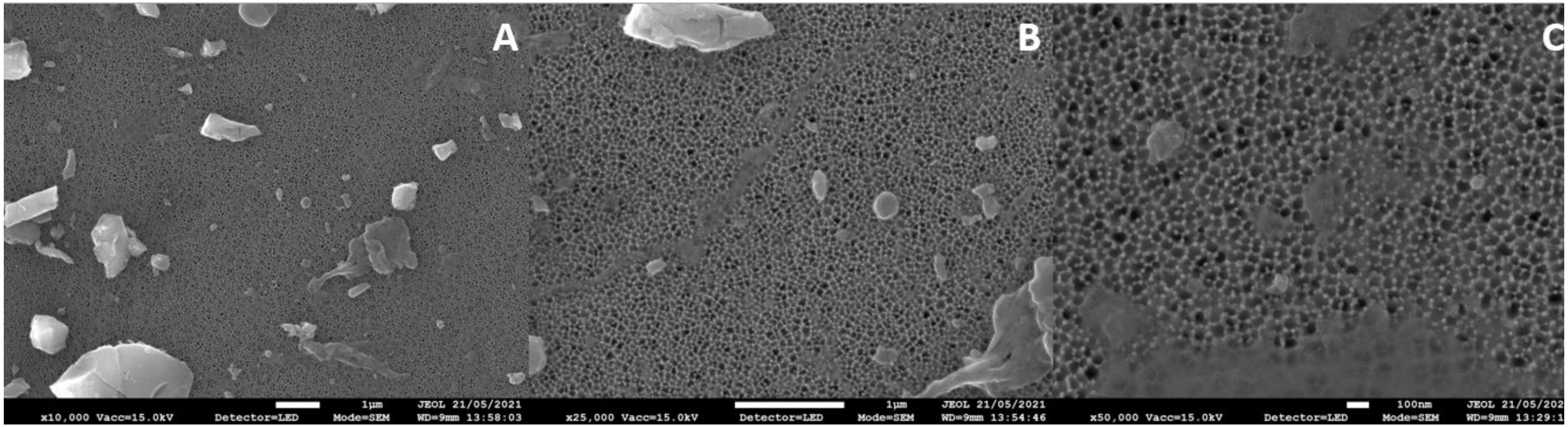


Figure 38S. FEG-SEM replicates of 0.02 µm pore size membranes for Brand NHS (IIR). (A) 10000x, (B) 25000x and (C) 50000x.

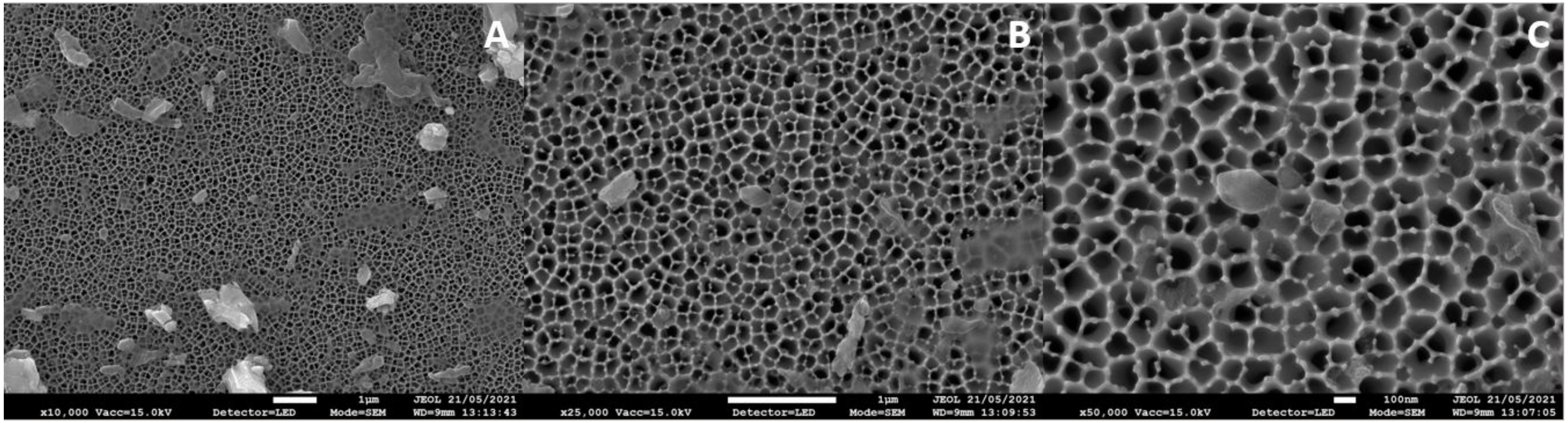


Figure 39S. FEG-SEM replicates of 0.1 µm pore size membranes for Brand NHS (IIR). (A) 10000x, (B) 25000x and (C) 50000x.

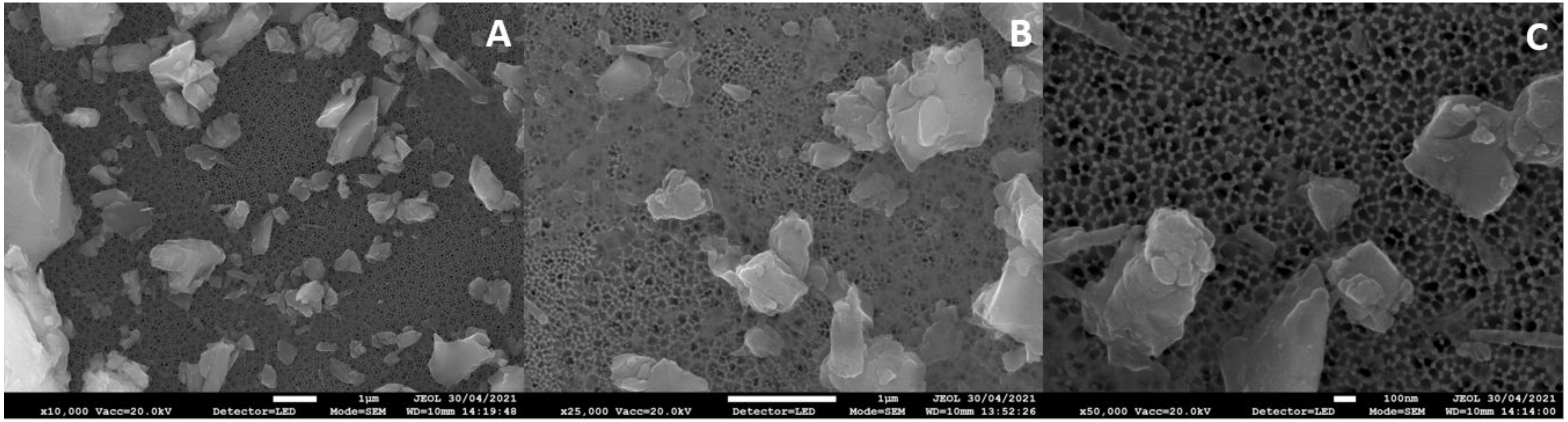


Figure 40S. FEG-SEM replicates of 0.02 µm pore size membranes for Brand Omnitex (IIR). (A) 10000x, (B) 25000x and (C) 50000x.

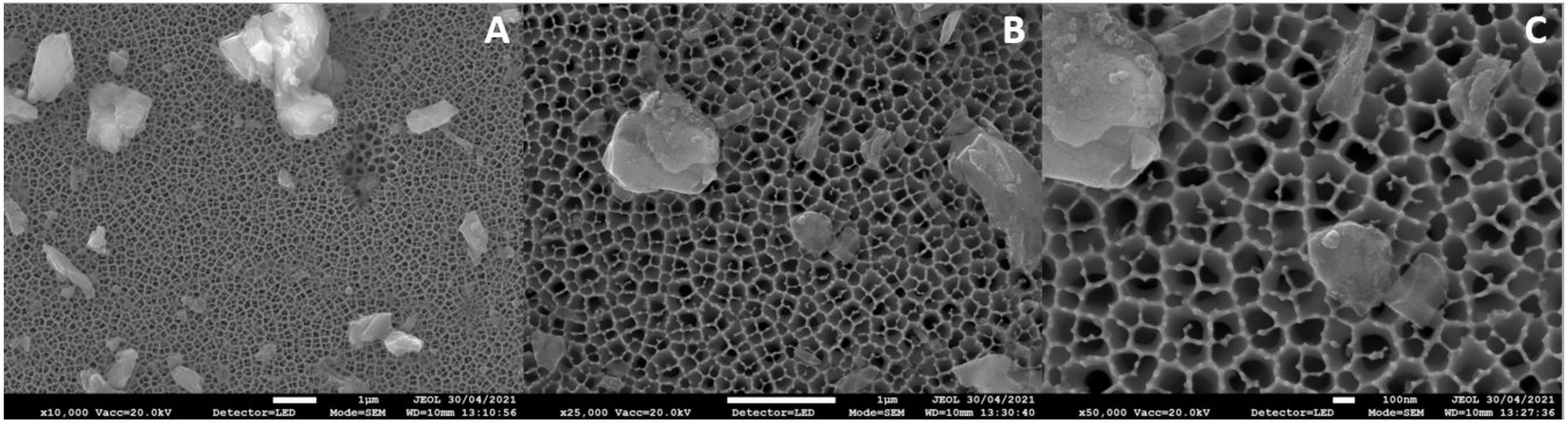


Figure 41S. FEG-SEM replicates of 0.1 µm pore size membranes for Brand Omnitex (IIR). (A) 10000x, (B) 25000x and (C) 50000x.

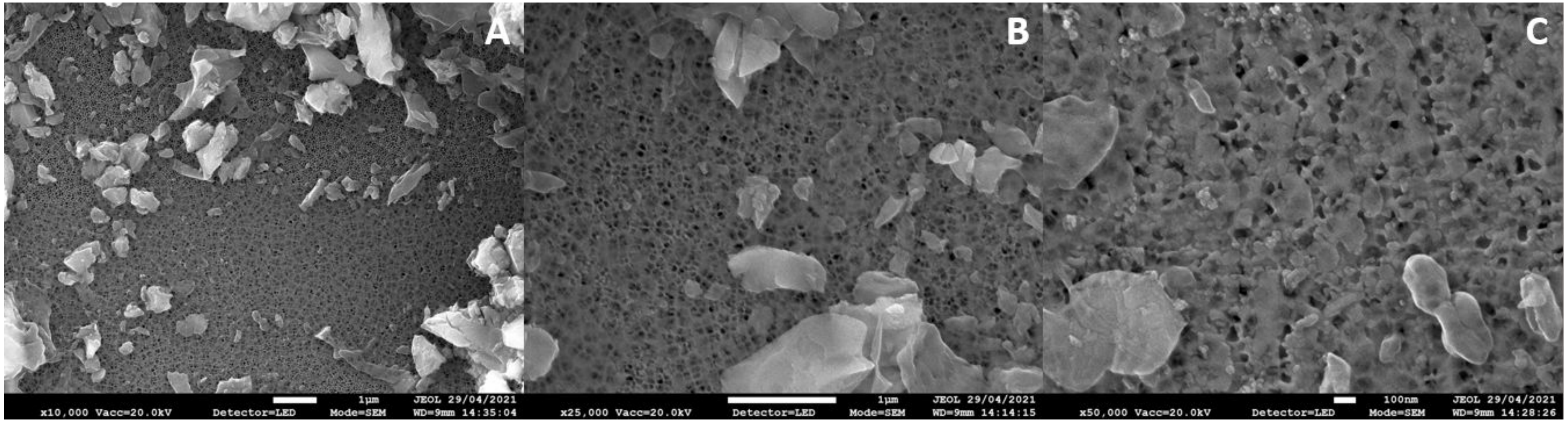


Figure 42S. FEG-SEM replicates of 0.02 µm pore size membranes for Brand Soyes (FFP2). (A) 10000x, (B) 25000x and (C) 50000x.

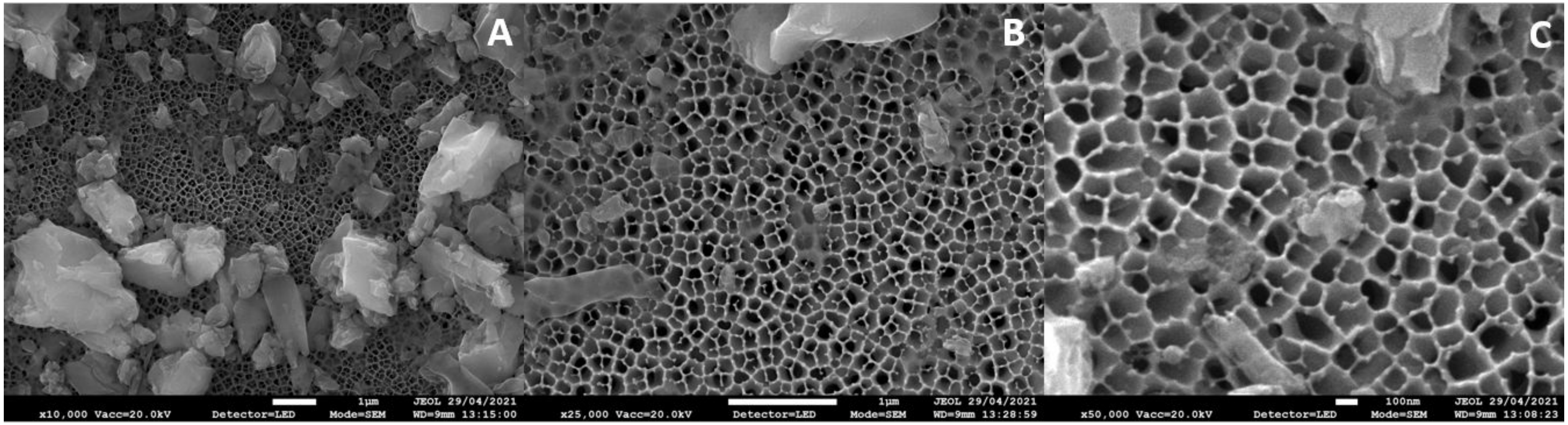


Figure 43S. FEG-SEM replicates of 0.1 µm pore size membranes for Brand Soyes (FFP2). (A) 10000x, (B) 25000x and (C) 50000x.

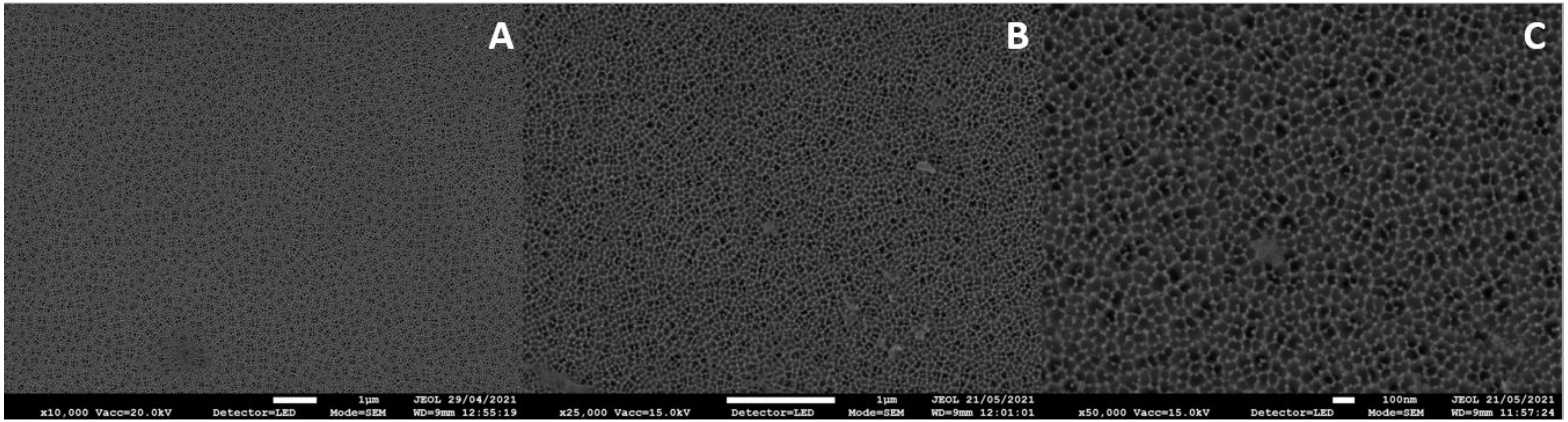


Figure 44S. FEG-SEM replicates of 0.02 µm pore size membranes Blank samples. (A) 10000x, (B) 25000x and (C) 50000x.

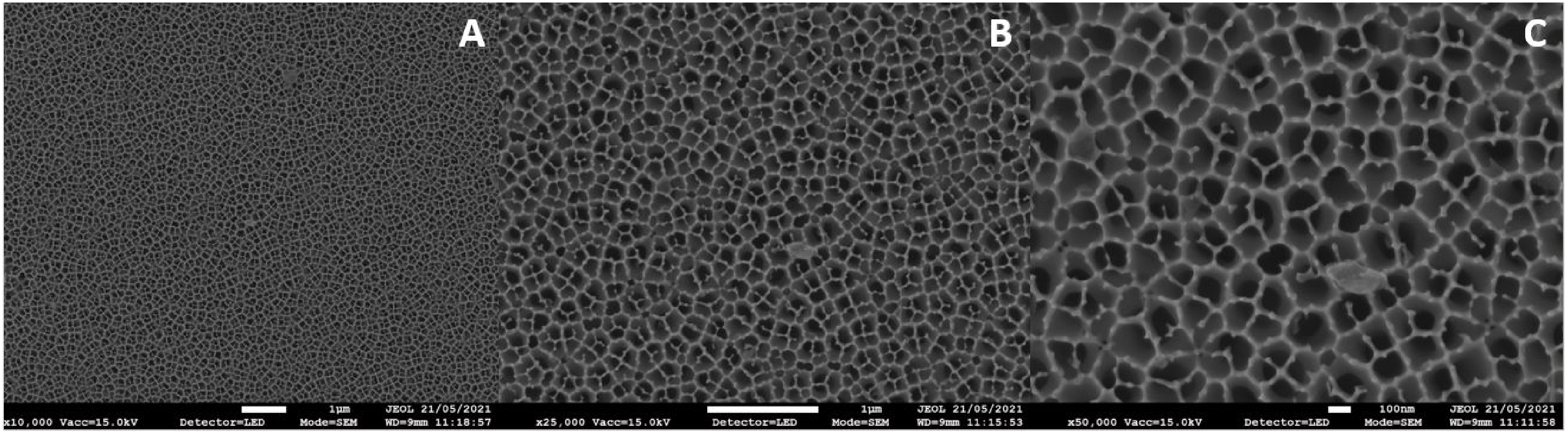


Figure 45S. FEG-SEM replicates of 0.1 µm pore size membranes Blank samples. (A) 10000x, (B) 25000x and (C) 50000x.

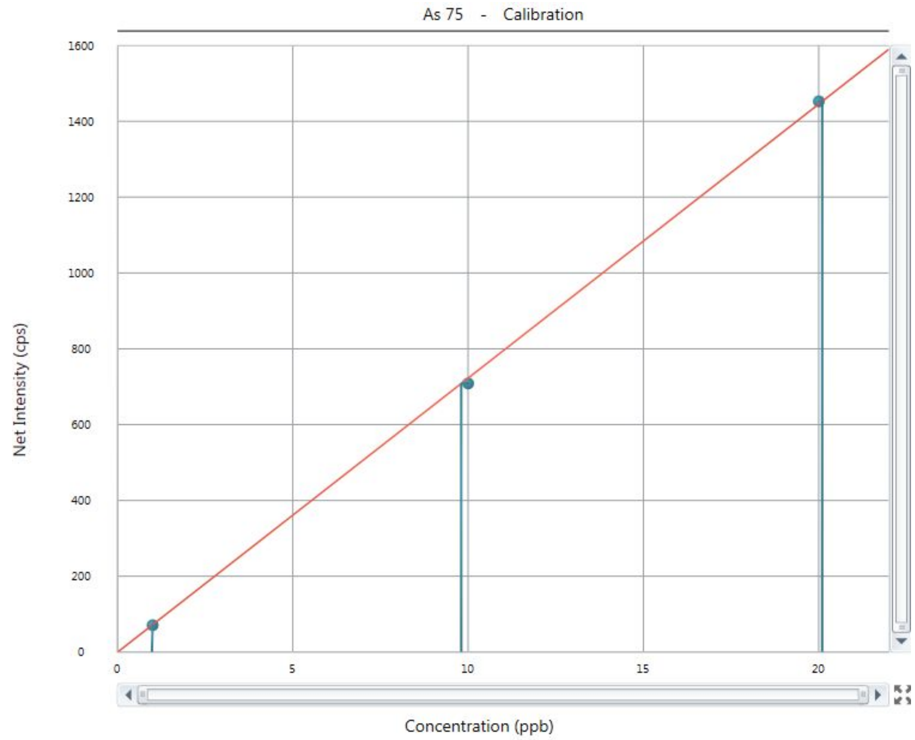


**Statistics**

| | | |
|---|---|---|
| Eqn. : | y = 72x + 0 | |
| Cor.Coeff. : | 0.999952 | |
| BEC : | 0.059951 | ppb |
| DL : | 0.047926 | ppb |

**Calibration Table**

| | Net Intensity As 75 (cps) | Apparent Conc. As 75 (ppb) |
|---|---|---|
| Blank | 4.3 | |
| Cal. Std.1 | 71.0 | 1.0 |
| Cal. Std.2 | 708.7 | 9.8 |
| Cal. Std.3 | 1452.7 | 20.1 |

Figure 46S - Calibration curve of the ICP-MS analyses of the leachates for As element.

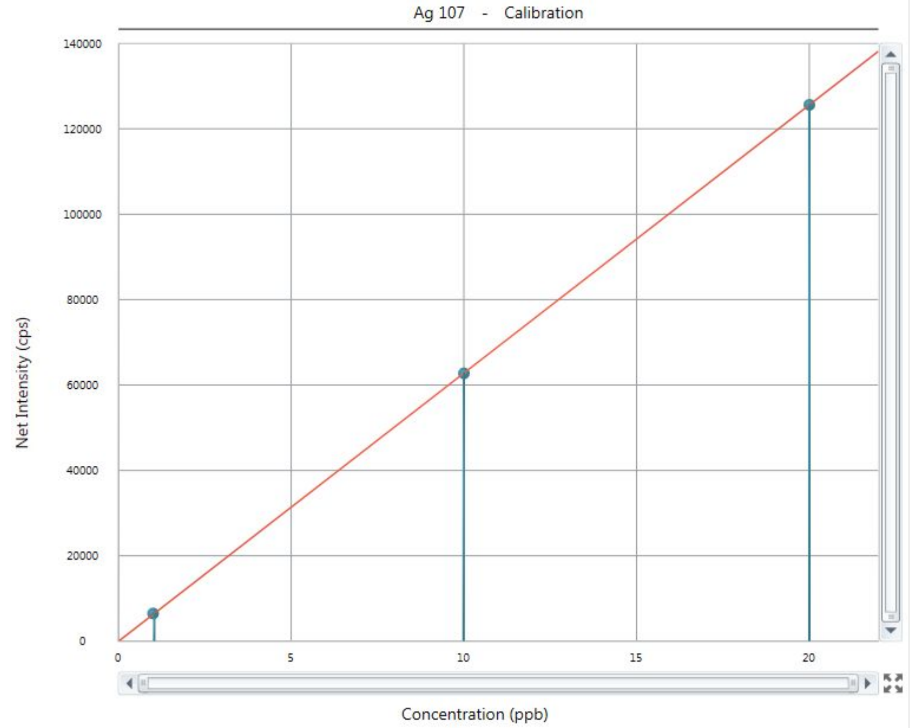


**Statistics**

| | | |
|---|---|---|
| Eqn. : | y = 6281x + 0 | |
| Cor.Coeff. : | 0.999999 | |
| BEC : | 0.177326 | ppb |
| DL : | 0.006761 | ppb |

**Calibration Table**

| | Net Intensity Ag 107 (cps) | Apparent Conc. Ag 107 (ppb) |
|---|---|---|
| Blank | 1113.7 | |
| Cal. Std.1 | 6505.7 | 1.0 |
| Cal. Std.2 | 62748.4 | 10.0 |
| Cal. Std.3 | 125629.2 | 20.0 |

Figure 47S - Calibration curve of the ICP-MS analyses of the leachates for Ag element.

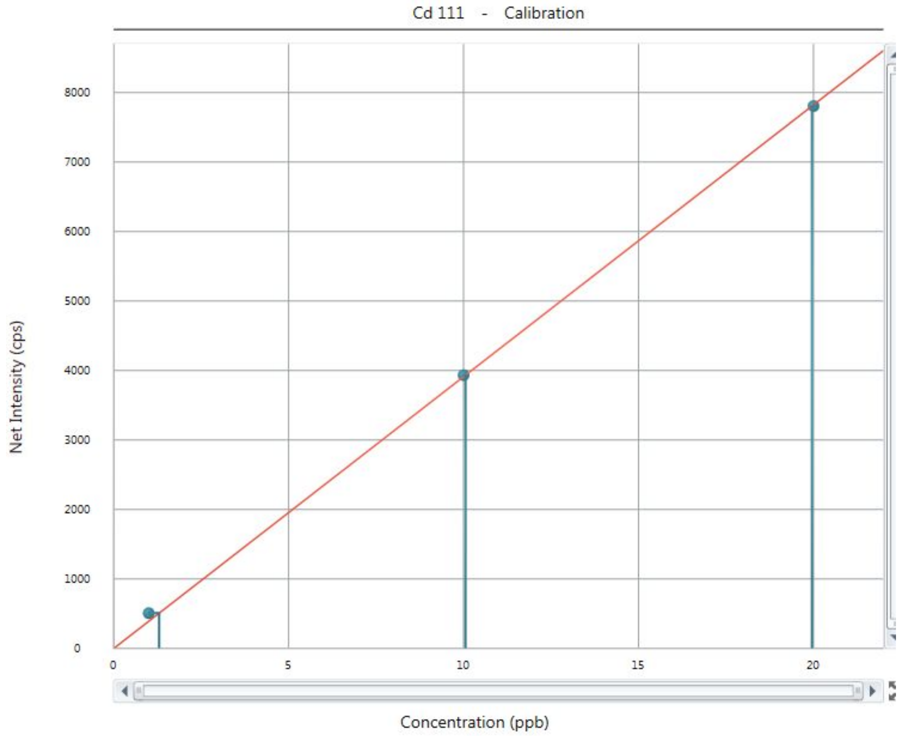


**Statistics**

| | |
|---|---|
| Eqn. : | y = 391x + 0 |
| Cor.Coeff. : | 0.999906 |
| BEC : | 0.094586 ppb |
| DL : | 0.033429 ppb |

**Calibration Table**

| | Net Intensity Cd 111 (cps) | Apparent Conc. Cd 111 (ppb) |
|---|---|---|
| Blank | 37.0 | |
| Cal. Std.1 | 508.3 | 1.3 |
| Cal. Std.2 | 3933.6 | 10.1 |
| Cal. Std.3 | 7806.8 | 20.0 |

Figure 48S - Calibration curve of the ICP-MS analyses of the leachates for Cd element.

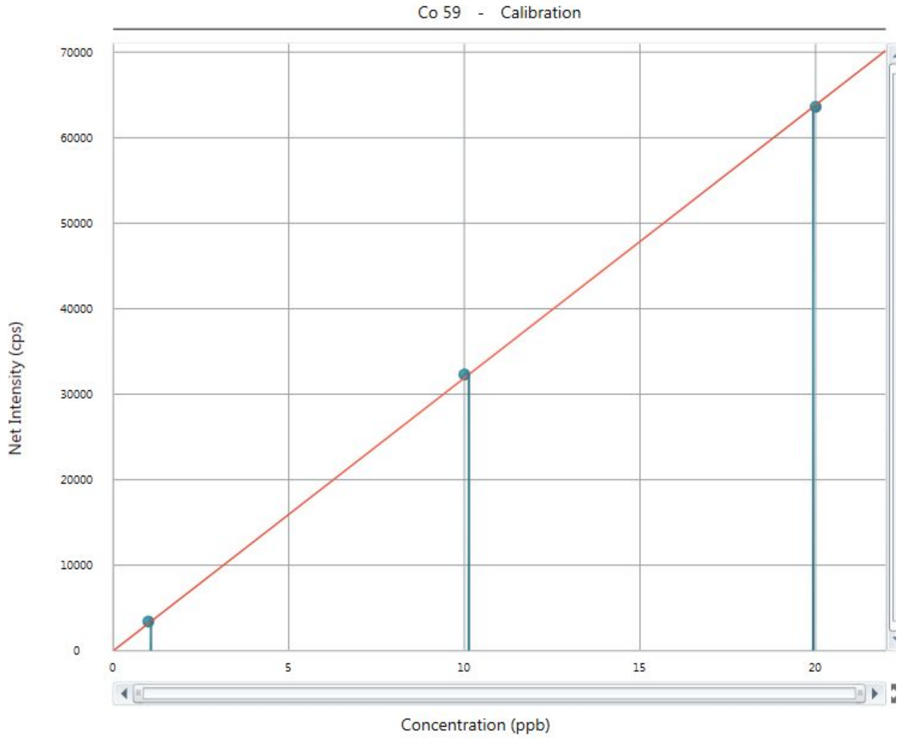


**Statistics**

| | |
|---|---|
| Eqn. : | y = 3191x + 0 |
| Cor.Coeff. : | 0.999974 |
| BEC : | 0.022353 ppb |
| DL : | 0.011333 ppb |

**Calibration Table**

| | Net Intensity Co 59 (cps) | Apparent Conc. Co 59 (ppb) |
|---|---|---|
| Blank | 71.3 | |
| Cal. Std.1 | 3424.1 | 1.1 |
| Cal. Std.2 | 32314.7 | 10.1 |
| Cal. Std.3 | 63610.0 | 19.9 |

Figure 49S - Calibration curve of the ICP-MS analyses of the leachates for Co element.

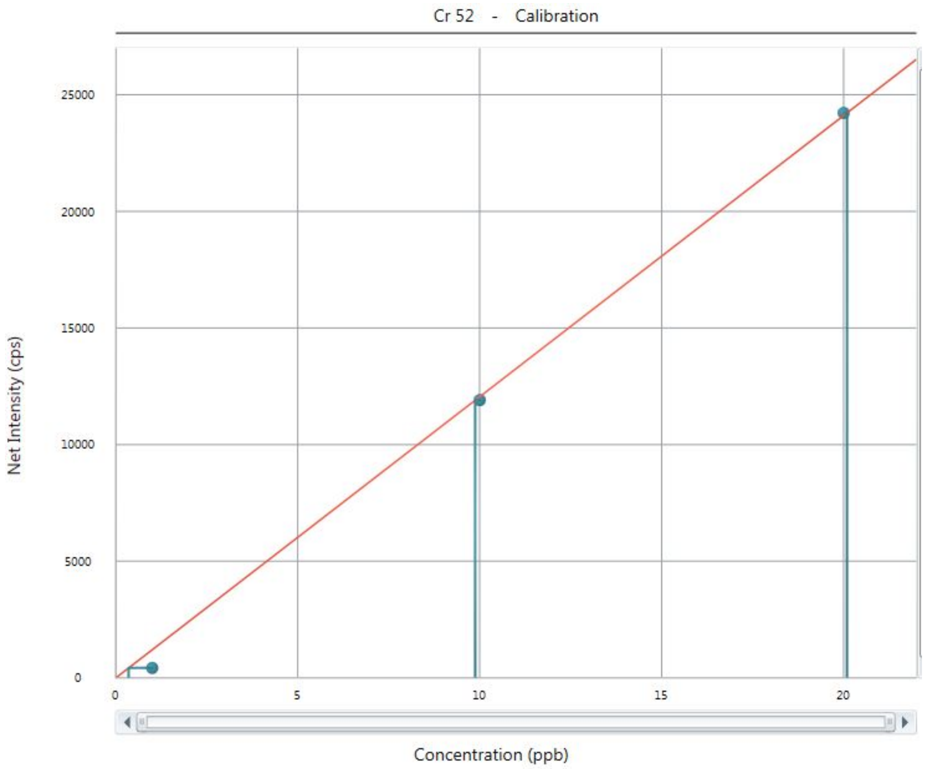


**Statistics**

| | | |
|---|---|---|
| Eqn. : | y = 1206x + 0 | |
| Cor.Coeff. : | 0.999561 | |
| BEC : | 0.983233 | ppb |
| DL : | 0.051264 | ppb |

**Calibration Table**

| | Net Intensity Cr 52 (cps) | Apparent Conc. Cr 52 (ppb) |
|---|---|---|
| Blank | 1185.4 | |
| Cal. Std.1 | 427.4 | 0.4 |
| Cal. Std.2 | 11909.3 | 9.9 |
| Cal. Std.3 | 24224.2 | 20.1 |

Figure 50S - Calibration curve of the ICP-MS analyses of the leachates for Cr element.

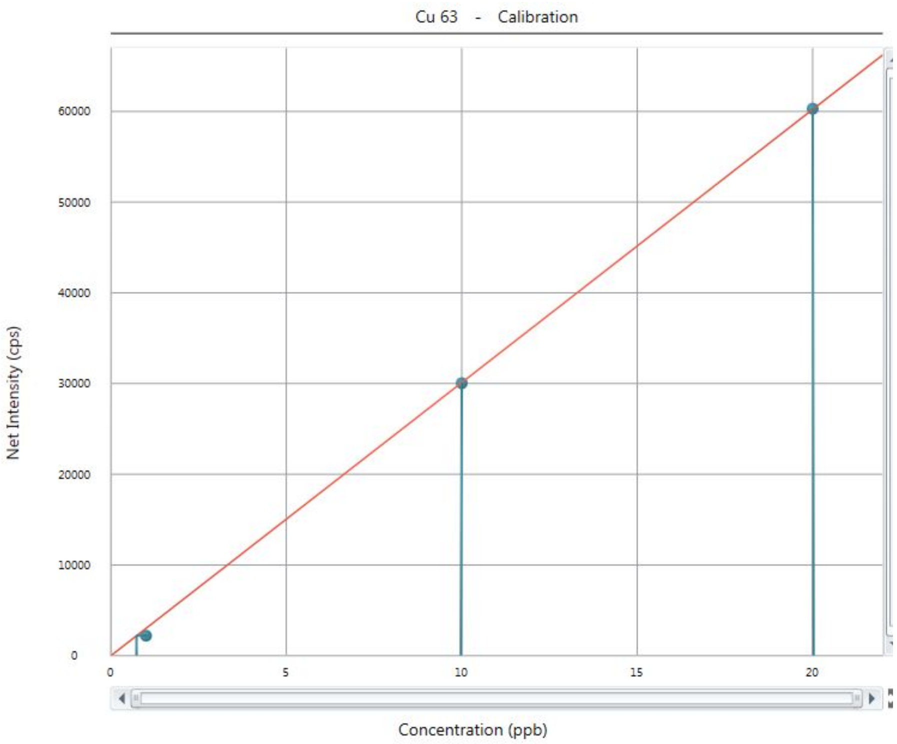


**Statistics**

| | |
|---|---|
| Eqn. : | y = 3011x + 0 |
| Cor.Coeff. : | 0.999927 |
| BEC : | 0.669418 ppb |
| DL : | 0.176815 ppb |

**Calibration Table**

| | Net Intensity Cu 63 (cps) | Apparent Conc. Cu 63 (ppb) |
|---|---|---|
| Blank | 2015.8 | |
| Cal. Std.1 | 2205.1 | 0.7 |
| Cal. Std.2 | 30040.5 | 10.0 |
| Cal. Std.3 | 60302.2 | 20.0 |

Figure 51S - Calibration curve of the ICP-MS analyses of the leachates for Cu element.

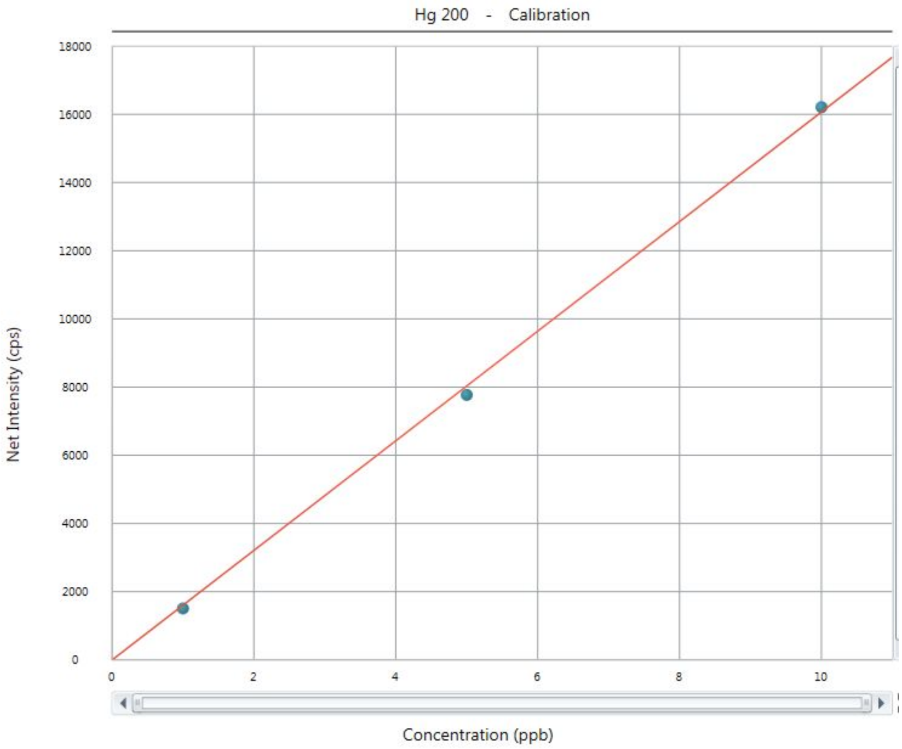


**Statistics**

| | |
|---|---|
| Eqn. : | y = 1607x + 0 |
| Cor.Coeff. : | 0.999850 |
| BEC : | 0.165417 ppb |
| DL : | 0.014135 ppb |

**Calibration Table**

| | Net Intensity Hg 200 (cps) | Apparent Conc. Hg 200 (ppb) |
|---|---|---|
| Blank | 265.8 | |
| Cal. Std.1 | 1511.1 | 0.9 |
| Cal. Std.2 | 7773.3 | 4.8 |
| Cal. Std.3 | 16211.3 | 10.1 |

Figure 52S - Calibration curve of the ICP-MS analyses of the leachates for Hg element.

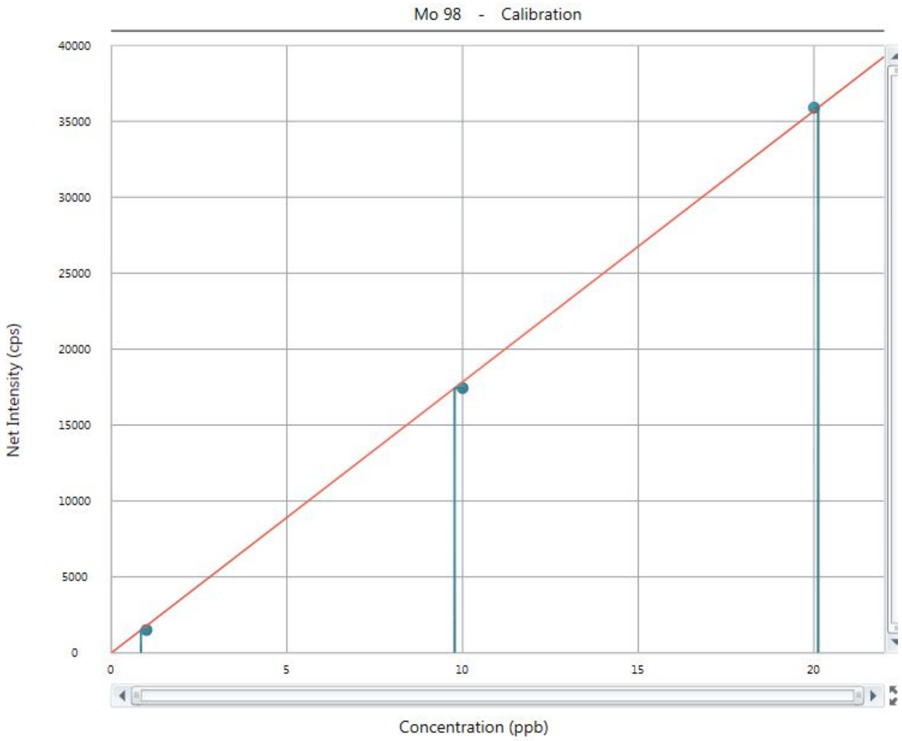


**Statistics**

| | | |
|---|---|---|
| Eqn. : | y = 1784x + 0 | |
| Cor.Coeff. : | 0.999912 | |
| BEC : | 0.048699 | ppb |
| DL : | 0.004238 | ppb |

**Calibration Table**

| | Net Intensity Mo 98 (cps) | Apparent Conc. Mo 98 (ppb) |
|---|---|---|
| Blank | 86.9 | |
| Cal. Std.1 | 1512.7 | 0.8 |
| Cal. Std.2 | 17441.4 | 9.8 |
| Cal. Std.3 | 35903.1 | 20.1 |

Figure 53S - Calibration curve of the ICP-MS analyses of the leachates for Mo element.

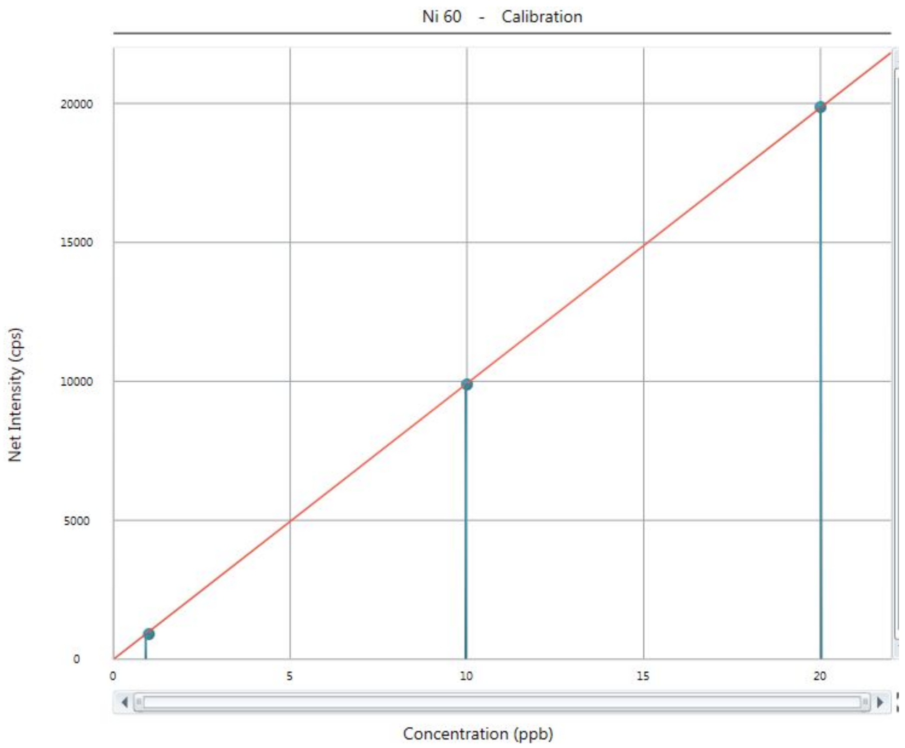


**Statistics**

| | | |
|---|---|---|
| Eqn. : | y = 992x + 0 | |
| Cor.Coeff. : | 0.999991 | |
| BEC : | 0.070196 | ppb |
| DL : | 0.006981 | ppb |

**Calibration Table**

| | Net Intensity Ni 60 (cps) | Apparent Conc. Ni 60 (ppb) |
|---|---|---|
| Blank | 69.7 | |
| Cal. Std.1 | 906.7 | 0.9 |
| Cal. Std.2 | 9889.5 | 10.0 |
| Cal. Std.3 | 19871.2 | 20.0 |

Figure 54S - Calibration curve of the ICP-MS analyses of the leachates for Ni element.

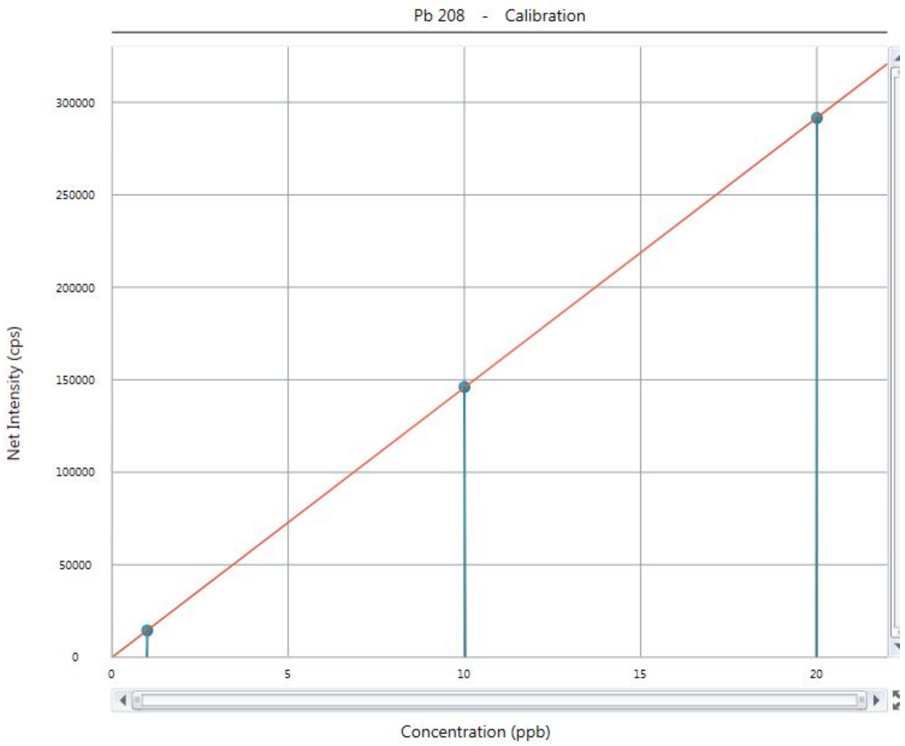


**Statistics**

| | | |
|---|---|---|
| Eqn. : | y = 14586x + 0 | |
| Cor.Coeff. : | 0.999999 | |
| BEC : | 0.242139 | ppb |
| DL : | 0.015799 | ppb |

**Calibration Table**

| | Net Intensity Pb 208 (cps) | Apparent Conc. Pb 208 (ppb) |
|---|---|---|
| Blank | 3531.8 | |
| Cal. Std.1 | 14506.3 | 1.0 |
| Cal. Std.2 | 146150.2 | 10.0 |
| Cal. Std.3 | 291571.6 | 20.0 |

Figure 55S - Calibration curve of the ICP-MS analyses of the leachates for Pb element.

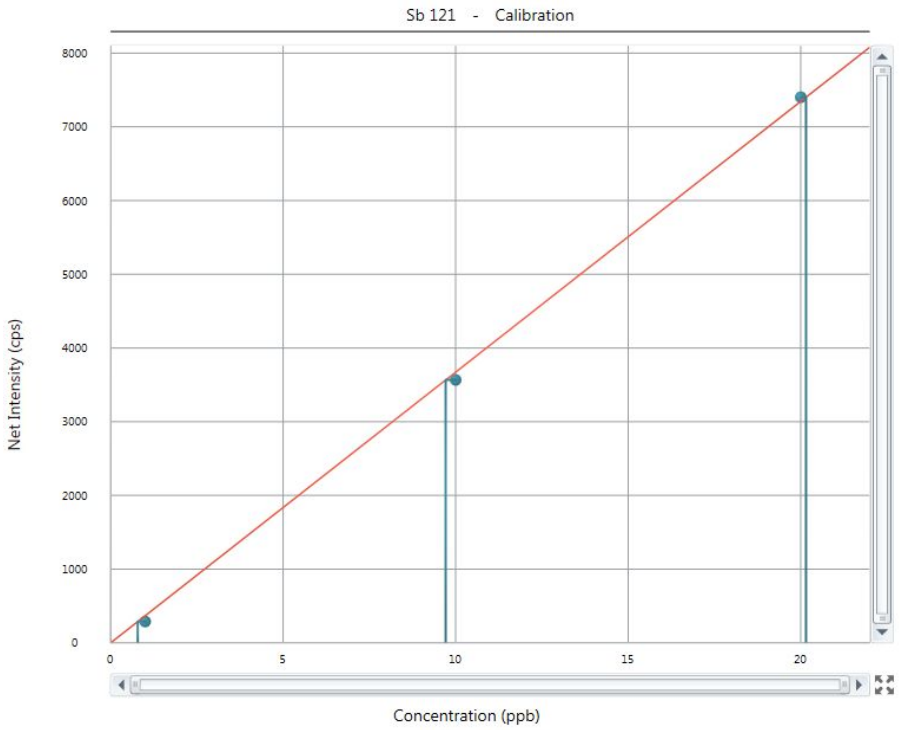


**Statistics**

| | |
|---|---|
| Eqn. : | y = 367x + 0 |
| Cor.Coeff. : | 0.999848 |
| BEC : | 0.463755 ppb |
| DL : | 0.111496 ppb |

**Calibration Table**

| | Net Intensity Sb 121 (cps) | Apparent Conc. Sb 121 (ppb) |
|---|---|---|
| Blank | 170.3 | |
| Cal. Std.1 | 289.3 | 0.8 |
| Cal. Std.2 | 3567.2 | 9.7 |
| Cal. Std.3 | 7402.7 | 20.2 |

Figure 56S - Calibration curve of the ICP-MS analyses of the leachates for Sb element.

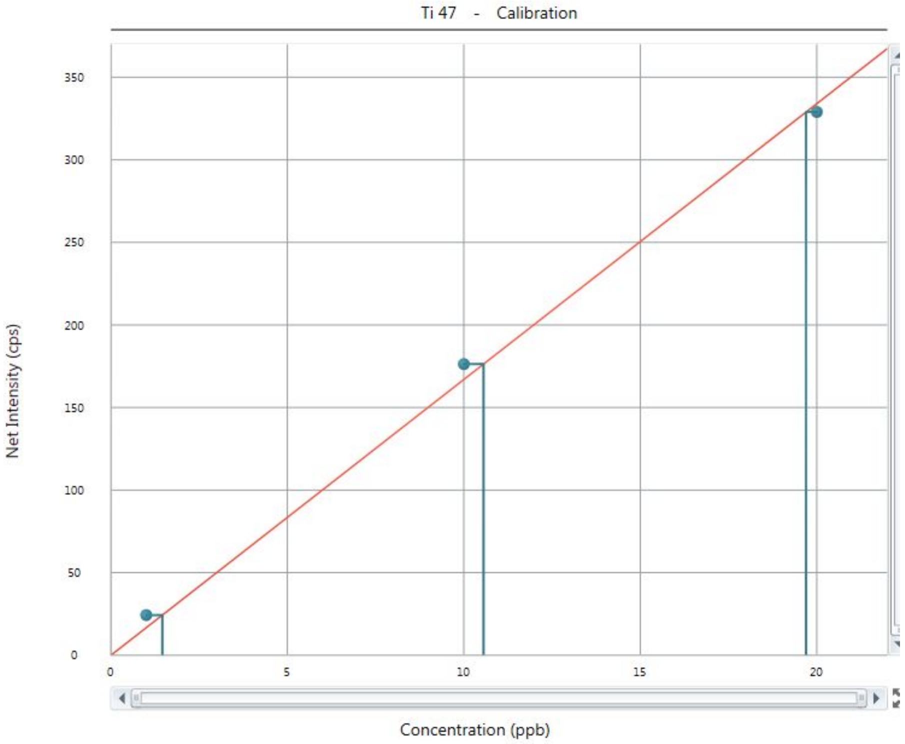


**Statistics**

| | |
|---|---|
| Eqn. : | y = 17x + 0 |
| Cor.Coeff. : | 0.999391 |
| BEC : | 0.179618 ppb |
| DL : | 0.311108 ppb |

**Calibration Table**

| | Net Intensity Ti 47 (cps) | Apparent Conc. Ti 47 (ppb) |
|---|---|---|
| Blank | 3.0 | |
| Cal. Std.1 | 24.3 | 1.5 |
| Cal. Std.2 | 176.3 | 10.6 |
| Cal. Std.3 | 329.0 | 19.7 |

Figure 57S - Calibration curve of the ICP-MS analyses of the leachates for Ti element.

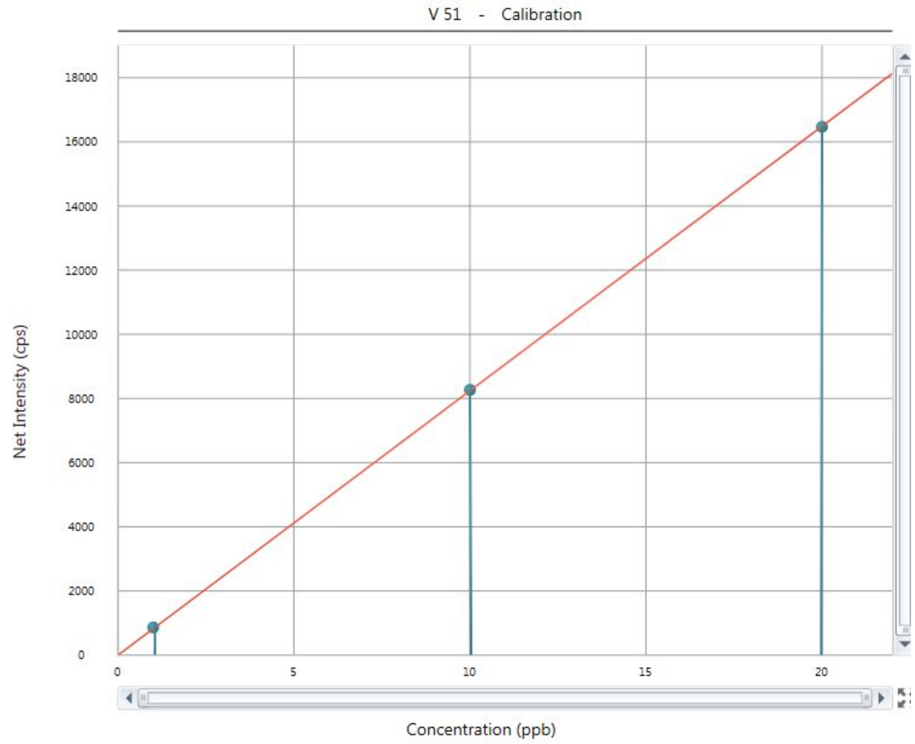


**Statistics**

| | | |
|---|---|---|
| Eqn. : | y = 824x + 0 | |
| Cor.Coeff. : | 0.999996 | |
| BEC : | 0.079263 | ppb |
| DL : | 0.033025 | ppb |

**Calibration Table**

| | Net Intensity V 51 (cps) | Apparent Conc. V 51 (ppb) |
|---|---|---|
| Blank | 65.3 | |
| Cal. Std.1 | 864.7 | 1.0 |
| Cal. Std.2 | 8269.4 | 10.0 |
| Cal. Std.3 | 16469.9 | 20.0 |

Figure 58S - Calibration curve of the ICP-MS analyses of the leachates for V element.

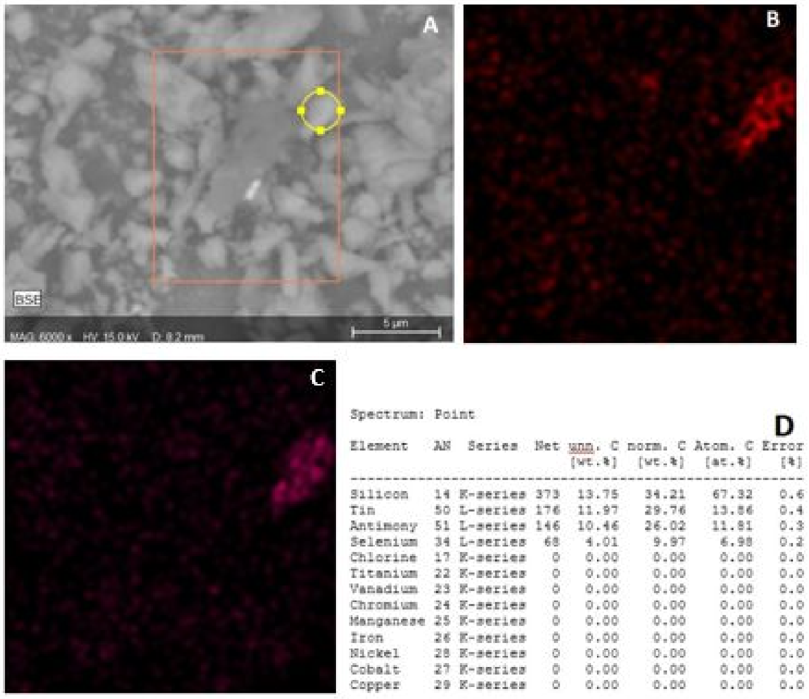


Spectrum: Point

| Element | AN | Series | Net | unn. C [wt.%] | norm. C [wt.%] | Atom. C [at.%] | Error [%] |
|---|---|---|---|---|---|---|---|
| Silicon | 14 | K-series | 373 | 13.75 | 34.21 | 67.32 | 0.6 |
| Tin | 50 | L-series | 176 | 11.97 | 29.76 | 13.86 | 0.4 |
| Antimony | 51 | L-series | 146 | 10.46 | 26.02 | 11.81 | 0.3 |
| Selenium | 34 | L-series | 68 | 4.01 | 9.97 | 6.98 | 0.2 |
| Chlorine | 17 | K-series | 0 | 0.00 | 0.00 | 0.00 | 0.0 |
| Titanium | 22 | K-series | 0 | 0.00 | 0.00 | 0.00 | 0.0 |
| Vanadium | 23 | K-series | 0 | 0.00 | 0.00 | 0.00 | 0.0 |
| Chromium | 24 | K-series | 0 | 0.00 | 0.00 | 0.00 | 0.0 |
| Manganese | 25 | K-series | 0 | 0.00 | 0.00 | 0.00 | 0.0 |
| Iron | 26 | K-series | 0 | 0.00 | 0.00 | 0.00 | 0.0 |
| Nickel | 28 | K-series | 0 | 0.00 | 0.00 | 0.00 | 0.0 |
| Cobalt | 27 | K-series | 0 | 0.00 | 0.00 | 0.00 | 0.0 |
| Copper | 29 | K-series | 0 | 0.00 | 0.00 | 0.00 | 0.0 |

Figure 59S. EDX data referring to the composition of a particle found in Baltic face mask. (A) is the image generated by the SEM at x6000, (B) is a false colour map for elemental Sb (C) is a false colour map for elemental Sn and (D) is the tabulated elemental composition data.

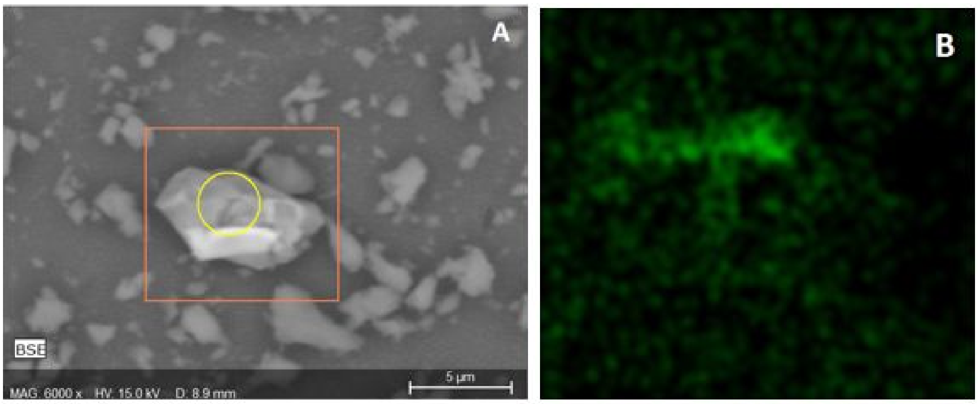


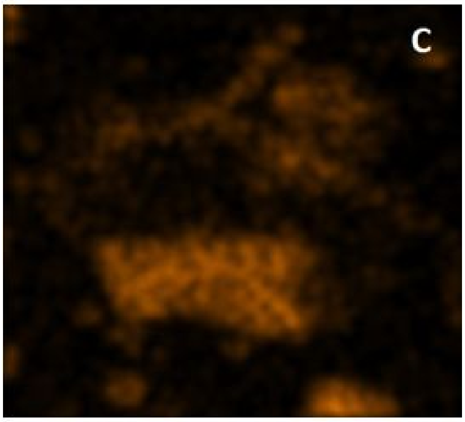


D

Spectrum: Point

| Element | AN | Series | Net | unn. C [wt.%] | norm. C [wt.%] | Atom. C [at.%] | Error [%] |
|---|---|---|---|---|---|---|---|
| Oxygen | 8 | K-series | 896 | 16.56 | 36.28 | 50.09 | 2.8 |
| Carbon | 6 | K-series | 188 | 7.80 | 17.08 | 31.40 | 1.9 |
| Silicon | 14 | K-series | 751 | 4.96 | 10.86 | 8.54 | 0.2 |
| Selenium | 34 | L-series | 420 | 4.26 | 9.34 | 2.61 | 0.2 |
| Arsenic | 33 | L-series | 343 | 3.57 | 7.83 | 2.31 | 0.2 |
| Iron | 26 | K-series | 124 | 2.57 | 5.63 | 2.23 | 0.1 |
| Tellurium | 52 | L-series | 247 | 3.12 | 6.83 | 1.18 | 0.1 |
| Manganese | 25 | K-series | 40 | 0.66 | 1.45 | 0.58 | 0.0 |
| Nickel | 28 | K-series | 9 | 0.32 | 0.69 | 0.26 | 0.0 |
| Antimony | 51 | L-series | 49 | 0.58 | 1.26 | 0.23 | 0.0 |
| Chromium | 24 | K-series | 13 | 0.17 | 0.38 | 0.16 | 0.0 |
| Copper | 29 | K-series | 4 | 0.18 | 0.40 | 0.14 | 0.0 |
| Cadmium | 48 | L-series | 18 | 0.19 | 0.41 | 0.08 | 0.0 |
| Mercury | 80 | M-series | 25 | 0.31 | 0.69 | 0.08 | 0.0 |
| Lead | 82 | M-series | 22 | 0.29 | 0.63 | 0.07 | 0.0 |
| Tin | 50 | L-series | 10 | 0.12 | 0.26 | 0.05 | 0.0 |
| | | | Total: | 45.66 | 100.00 | 100.00 | |

Figure 60S. EDX data referring to the composition of a particle found in Omnitex face mask. (A) is the image generated by the SEM at x6000, (B) is a false colour map for elemental As (C) is a false colour map for elemental Si and (D) is the tabulated elemental composition data.

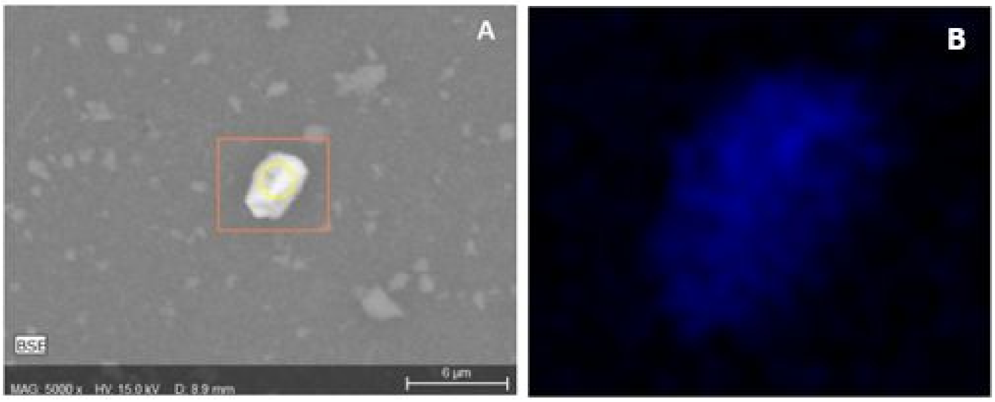


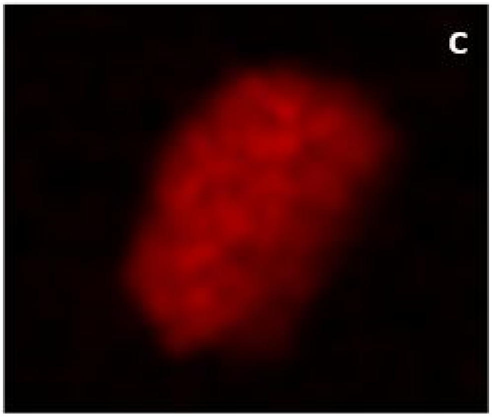


D

Spectrum: Point

| Element | AN | Series | Net | unn. C [wt.%] | norm. C [wt.%] | Atom. C [at.%] |
|---|---|---|---|---|---|---|
| Copper | 29 | K-series | 67 | 44.57 | 36.66 | 55.46 |
| Lead | 82 | M-series | 197 | 33.88 | 27.87 | 12.93 |
| Selenium | 34 | L-series | 145 | 23.21 | 19.09 | 23.24 |
| Mercury | 80 | M-series | 110 | 18.20 | 14.97 | 7.18 |
| Cadmium | 48 | L-series | 5 | 0.83 | 0.68 | 0.59 |
| Antimony | 51 | L-series | 4 | 0.82 | 0.67 | 0.53 |
| Manganese | 25 | K-series | 0 | 0.03 | 0.02 | 0.04 |
| Vanadium | 23 | K-series | 0 | 0.01 | 0.01 | 0.01 |
| Scandium | 21 | K-series | 0 | 0.00 | 0.00 | 0.00 |
| Titanium | 22 | K-series | 0 | 0.00 | 0.00 | 0.00 |
| Chromium | 24 | K-series | 0 | 0.00 | 0.00 | 0.00 |
| Iron | 26 | K-series | 0 | 0.00 | 0.00 | 0.00 |
| Cobalt | 27 | K-series | 0 | 0.00 | 0.00 | 0.00 |
| Nickel | 28 | K-series | 0 | 0.00 | 0.00 | 0.00 |
| Zinc | 30 | K-series | 0 | 0.00 | 0.00 | 0.00 |
| Silver | 47 | L-series | 0 | 0.00 | 0.00 | 0.00 |
| Rhodium | 45 | L-series | 0 | 0.00 | 0.00 | 0.00 |

Figure 61S. EDX data referring to the composition of a particle found in NHS face mask. (A) is the image generated by the SEM at x5000, (B) is a false colour map for elemental Hg. (C) is a false colour map for elemental Pb and (D) is the tabulated elemental composition data.

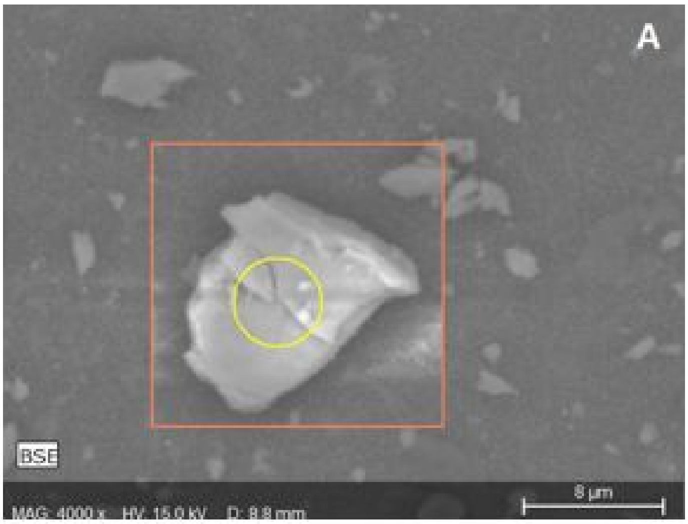


Spectrum: Point

**B**

| Element | AN | Series | Net | unn. C [wt.%] | norm. C [wt.%] | Atom. C [at.%] |
|---|---|---|---|---|---|---|
| Iron | 26 | K-series | 591 | 104.19 | 57.07 | 64.03 |
| Chromium | 24 | K-series | 81 | 8.57 | 4.69 | 5.65 |
| Selenium | 34 | L-series | 84 | 8.41 | 4.61 | 3.66 |
| Lead | 82 | M-series | 61 | 5.65 | 3.09 | 0.94 |
| Antimony | 51 | L-series | 54 | 4.92 | 2.69 | 1.39 |
| Mercury | 80 | M-series | 52 | 4.71 | 2.58 | 0.81 |
| Platinum | 78 | M-series | 50 | 4.60 | 2.52 | 0.81 |
| Cobalt | 27 | K-series | 18 | 4.24 | 2.32 | 2.47 |
| Nickel | 28 | K-series | 14 | 3.96 | 2.17 | 2.31 |
| Copper | 29 | K-series | 9 | 3.73 | 2.04 | 2.01 |
| Arsenic | 33 | L-series | 31 | 3.65 | 2.00 | 1.67 |
| Cadmium | 48 | L-series | 43 | 3.47 | 1.90 | 1.06 |
| Chlorine | 17 | K-series | 63 | 3.06 | 1.67 | 2.96 |
| Tellurium | 52 | L-series | 29 | 2.82 | 1.54 | 0.76 |
| Silicon | 14 | K-series | 48 | 2.58 | 1.41 | 3.15 |
| Tin | 50 | L-series | 26 | 2.26 | 1.24 | 0.65 |
| Palladium | 46 | L-series | 24 | 1.81 | 0.99 | 0.58 |
| Ruthenium | 44 | L-series | 23 | 1.70 | 0.93 | 0.58 |
| Manganese | 25 | K-series | 11 | 1.53 | 0.84 | 0.95 |
| Vanadium | 23 | K-series | 16 | 1.52 | 0.83 | 1.02 |
| Silver | 47 | L-series | 19 | 1.48 | 0.81 | 0.47 |
| Zinc | 30 | K-series | 2 | 1.32 | 0.72 | 0.69 |
| Titanium | 22 | K-series | 13 | 1.11 | 0.61 | 0.80 |

Figure 62S. EDX data referring to the composition of a complex particle from NHS face mask. (A) is the image generated by the SEM at x4000 and (B) is the tabulated elemental composition data.
